# Influence of Food Resource Availability on the Activity Time of Raccoon Dogs (*Nyctereutes procyonoides*) in Urban Parks in Tokyo

**DOI:** 10.1002/ece3.71966

**Published:** 2025-08-08

**Authors:** Shoya Sasaki, Takeshi Osawa

**Affiliations:** ^1^ Graduate School of Urban Environmental Sciences Tokyo Metropolitan University Hachiouji Tokyo Japan

**Keywords:** activity time, feeding habits, raccoon dogs, urban parks, urbanization

## Abstract

Urbanization results in the reduction and fragmentation of green spaces, which function as wildlife habitats. However, some wildlife has adapted to urbanization by changing their ecology and behaviors. The raccoon dog ( *Nyctereutes procyonoides* ) has established itself in urbanized environments due to its flexible ecology including activity time and feeding habits. However, there have been few studies on the mechanism of its adaptation to urbanized environments. In this study, we evaluated the relationship between raccoon dog activity time and food resources and the degree of urbanization in the western area of Tokyo, the most populated region of Japan. Results showed that raccoon dogs tend to increase their daytime activity and use artificial food resources according to the degree of urbanization, but this is seasonally dependent. This suggests that the activity time and feeding habits of raccoon dogs are linked to their adaptation to urbanized environments. Specifically, daytime activity may provide an advantage for access to artificial food resources. Therefore, with increasing urbanization, activity time and feeding habits may change. Given that wild mammals, such as raccoon dogs, may alter their activity time and feeding habits in response to urbanization and that these changes may be interrelated, examining activity time and feeding habits in conjunction is essential.

## Graphical abstract

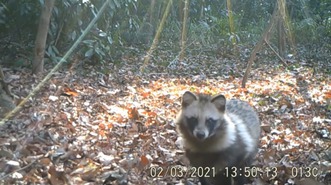

## 1 Introduction

In recent years, human populations have become increasingly concentrated in urban areas worldwide, resulting in the expansion of urbanized areas (Angel et al. 2005; Seto et al. 2012). This land cover change results in both reduced and fragmented green spaces that act as habitats for wildlife, leading to changes in community structures and a decline in biodiversity (Natuhara and Imai 1996; Grimm et al. 2008; Takaoka 2013). However, some studies have suggested that some wildlife successfully adapt to urbanized environments by changing their ecology and behaviors such as activity time and feeding habits (Baker and Harris 2007; Shochat et al. 2010; Lapiedra and González‐Lagos 2013).

Wild mammals often successfully adapt to urbanized environments when they offer suitable environmental conditions such as abundant food resources including a stable supply of food scraps, fewer predators, and anthropogenic landscapes that provide shelter (Bateman and Fleming 2012; Santini et al. 2019; Enari 2019). To utilize these conditions, wild mammals often change their ecology and behavior (Lapiedra and González‐Lagos 2013). The ability to adjust activity times and food resources to fit urbanized environments is crucial for the urban adaptation of wildlife (Ditchkoff et al. 2006). Some species have been observed to switch to total nocturnal activity or to reduce daytime activity to avoid humans, shifting from natural food resources to utilizing abundant artificial food resources in urban environments (Ditchkoff et al. 2006; Gehrt 2007; Lowry et al. 2013). For instance, the bobcats ( *Lynx rufus* ) and coyotes ( *Canis latrans* ) have adapted to urban environments by modifying their activity time from daytime to nighttime to avoid humans (Riley et al. 2003; Stark et al. 2020). European badgers ( *Meles meles* ) and red foxes ( *Vulpes vulpes* ) have adjusted to urban settings, and their populations have been increased by altering their food resources to anthropogenic food sources (Contesse et al. 2004; Gomes et al. 2020).

**FIGURE 1:**
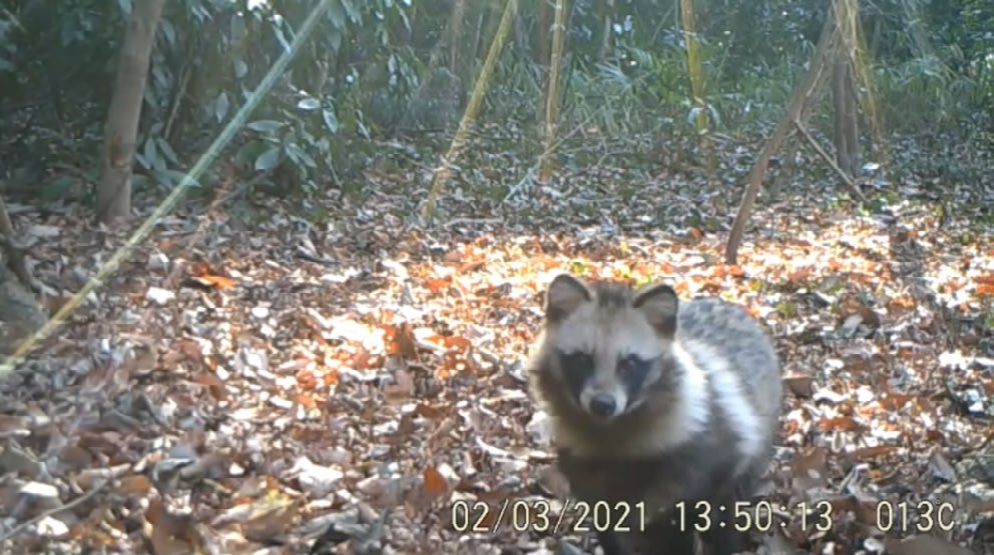
Raccoon dog photographed by a camera trap in Tokyo, Japan.

The raccoon dog ( *Nyctereutes procyonoides* ) (Figure 1), distributed throughout almost the whole of Japan, exhibits behavioral flexibility by opportunistically using various new resources such as food resources and activity sites (Sonoda and Kuramoto 2008). Although their typical habitat is *Satoyama*, which is a mixture of semi‐natural forests and farmlands, their flexible ecological characteristics enable their adaptation to urban environments (Ohdachi et al. 2015). Several studies have demonstrated that raccoon dogs inhabit urban environments (Saito and Koike 2013; Saito et al. 2018; Kamito et al. 2019; Enari 2019; Tsunoda et al. 2019; Wang et al. 2024). Additionally, raccoon dogs have become an invasive alien species in some parts of Europe, demonstrating their ability to adapt to a variety of environments (Kauhala and Kowalczyk 2011). However, there are few studies that tested the factors that allow raccoon dogs to adapt to urban environments.

Previous studies have indicated that shifts in activity time and dietary habits are important factors contributing to adaptation to the urban environments of mammals (Ditchkoff et al. 2006; Contesse et al. 2004; Gehrt 2007; Lowry et al. 2013). Raccoon dogs are nocturnal animals but are often observed to be active during the day (Okabe and Agetsuma 2007; Ikeda et al. 2016; Sasaki and Osawa 2022). In addition, they flexibly utilize food resources depending on the season and surrounding environment (Akihito et al. 2016; Sasaki and Osawa 2023). Therefore, we hypothesized that raccoon dogs adapt to urban environments by becoming more nocturnal and changing their feeding habits to depend on artificial food resources according to the degree of urbanization in their home ranges.

In this study, we surveyed raccoon dog activity time and feeding habits in eight urban parks with various degrees of urbanization in Tokyo, Japan. We aimed to evaluate the relationships between raccoon dog activity time, feeding habits, and the degree of urbanization in each park. We predicted that raccoon dogs would become completely nocturnal to avoid human activity and would shift their food resources from natural to artificial ones according to the degree of urbanization of each urban park. Based on the results, we discussed the adaptation mechanism of raccoon dogs to urban environments.

## 2 Materials and Methods

### 2.1 Study Sites

The study was conducted in the South‐Tama region and Setagaya Ward within Tokyo, Japan. Tokyo is the most populated prefecture in Japan. The South‐Tama region contains a wide range of residential areas with many urban parks and seminatural ecosystems including broadleaf forests and grasslands. There are relatively large green spaces because of urbanization in recent years. In stark contrast, Setagaya Ward has been urbanized for a long time; therefore, residential land occupies upward of 90% of the territorial expanse (Setagaya Ward 2016). Consequently, although vestiges of sylvan areas suitable for wildlife habitation endure within a confined spatial area of approximately 30–40 km, urban topography is dominant.

**FIGURE 2:**
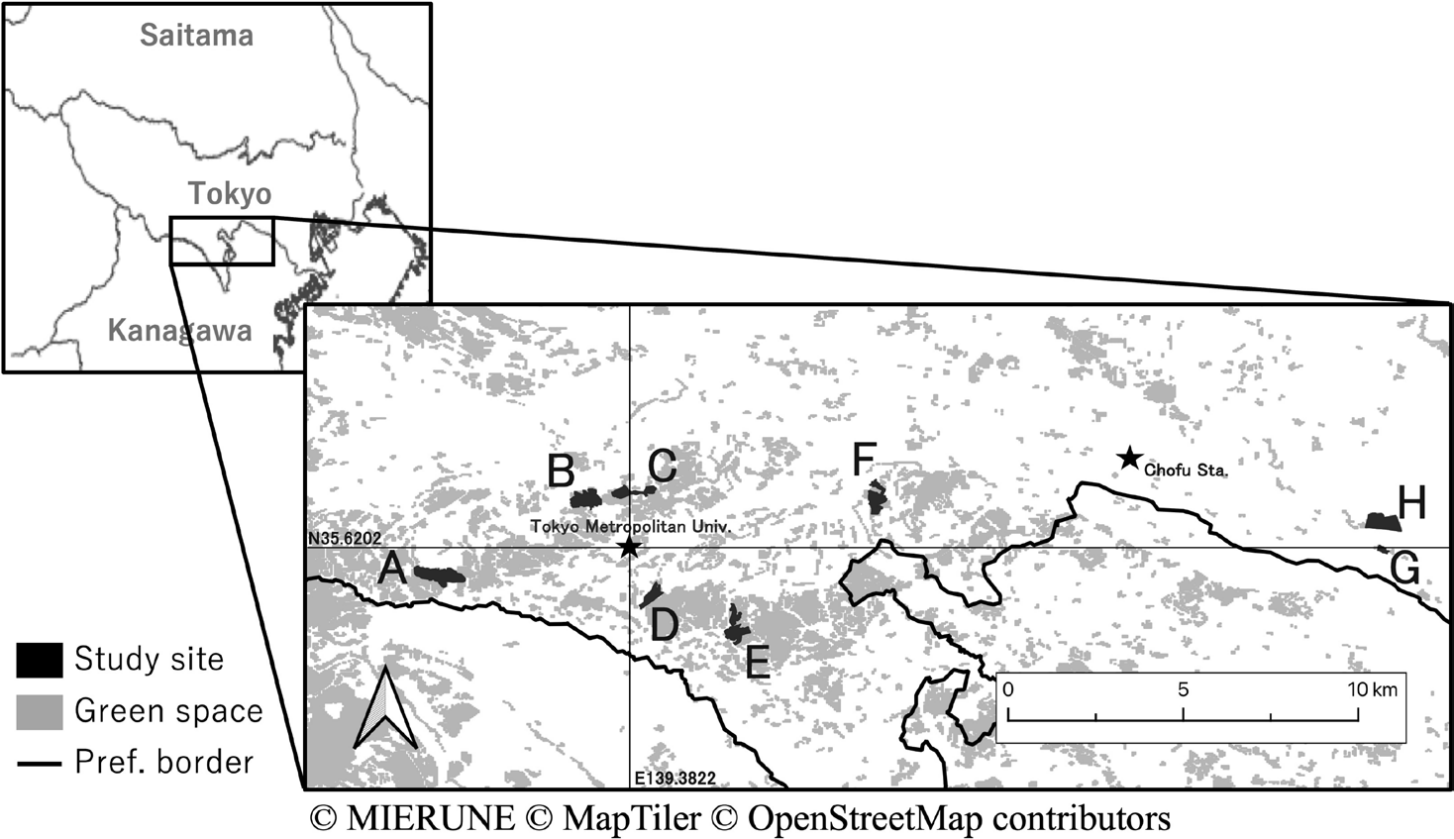
Study sites. The study sites are indicated by black, green spaces by gray, and the prefectural border by black lines. (A–H) Correspond to the IDs in Table 1 (A: Nanakuni Ridge Green Space, B: Naganuma Park, C: Hirayama Joshi Park, D: Nagaike Park, E: Oyamada Green Space, F: Sakuragaoka Park, G: Okamoto Seikado Green Space, H: Kinuta Park).

We selected eight urban parks that have different conditions for the surrounding landscape (i.e., degrees of urbanization): Nanakuni Ridge Green Space, Naganuma Park, Hirayama Joshi Park, Nagaike Park, Oyamada Green Space, Sakuragaoka Park, Okamoto Seikado Green Space, and Kinuta Park (Figure 2).

### 2.2 Landscape Structures of Each Urban Park

We calculated the artificial land cover area in both internal and external parks as an index of urbanization using the current vegetation maps derived from the 6th and 7th Natural Environment Surveys (Ministry of the Environment; https://www.biodic.go.jp/index_e.html, last accessed April 19, 2024). This data are part of the results of a nationwide natural environment survey conducted by the Ministry of the Environment of Japan under the Natural Environment Conservation Act (Ministry of the Environment; https://www.biodic.go.jp/index_e.html, last accessed April 19, 2024). We used the residential class in the vegetation map as an urban area. Prior to calculating the urban area, we overlaid the vegetation map and the most recent aerial photographs, captured in 2019, sourced from the Ministry of Land, Infrastructure, Transport and Tourism's repository of national photographs (latest national photographs https://maps.gsi.go.jp/development/ichiran.html#ort, last accessed on April 19, 2024) to define the borders of the urban parks. In areas exhibiting shifts in land cover or conspicuous alterations, we adjusted polygons to align with the current border.

We then established 500‐ and 1000‐m buffer polygons from the park borders and calculated the urban area and its proportion within the buffers. The spatial scale of 500 m is essential for the habitat of a raccoon dog (Saito and Koike 2013). Thus, we used 500 and 1000 m to evaluate the effects of urbanization.

Table 1 summarizes the landscape structures of urban parks, including the surrounding areas, as calculated using GIS analysis. The Nanakuni Ridge Green Area had the largest area (560,441 m^2^), whereas the Okamoto Seikado Green Area had the smallest area (42,212 m^2^). Kinuta Park contained the largest area of urban landscapes (22,233 m^2^), whereas Okamoto Seikado Greenspace contained the smallest area of urban landscapes (2445 m^2^) (Table 1). Within the 500 m buffer zones, Kinuta Park exhibited the most extensive urban landscapes (1,353,632 m^2^), whereas Nagaike Park exhibited the least extent of urban landscapes (401,843 m^2^) (Table 1). Similarly, within the 1000 m buffer zones, Kinuta Park exhibited the greatest area of anthropogenic landscapes (3,859,606 m^2^), whereas Nagaike Park exhibited the lowest area of anthropogenic landscapes (1,278,013 m^2^) (Table 1).

**TABLE 1:** Area (m^2^) of each land type and the number of cameras in each park.

ID	Park	Range	Forest	Urban	Others	Total	No. of cameras	No. of sighting for racoon dog	No. of sighting for racoon
A	Nanakuni Ridge Green Space	Within the park	513,489.8	11,239.4	35,711.8	**560,440.9**	4	162	245
		500 m buffer	921,464.4	578,041.4	518,040.5	2,017,546.3			
		1000 m buffer	1,933,907.2	1,889,617.8	1,175,441.8	4,998,966.8			
B	Naganuma Park	Within the park	376,310.3	6754.7	21,260.1	404,325.1	3	564	58
		500 m buffer	386,559.9	1,007,178.5	259,439.7	1,653,178.1			
		1000 m buffer	870,543.3	2,722,244.9	664,881.4	4,257,669.5			
C	Hirayama Joshi Park	Within the park	230,233.8	7901.1	14,072.0	252,207.9	3	610	58
		500 m buffer	729,078.3	849,398.9	228,152.6	1,806,629.8			
		1000 m buffer	1,716,707.5	2,036,344.7	792,338.5	4,545,390.7			
D	Nagaike Park	Within the park	147,442.1	14,523.6	44,646.6	206,612.3	3	231	60
		500 m buffer	468,168.4	401,843.2	640,667.4	1,510,679.0			
		1000 m buffer	980,602.5	1,278,012.8	1,748,592.2	4,007,207.5			
E	Oyamada Green Space	Within the park	313,817.8	4562.9	60,638.3	379,019.0	3	270	31
		500 m buffer	688,589.9	612,255.2	747,648.9	2,048,494.0			
		1000 m buffer	2,004,993.1	1,391,681.5	1,556,311.7	4,952,986.3			
F	Sakuragaoka Park	Within the park	269,775.4	13,234.2	55,650.4	338,660.0	4	809	1
		500 m buffer	274,494.7	1,176,042.6	343,606.4	1,794,143.7			
		1000 m buffer	810,238.3	2,388,665.6	1,292,825.5	4,491,729.5			
G	Okamoto Seikado Green Space	Within the park	39,766.4	2445.3	0.0	*42,211.8*	3	339	295
		500 m buffer	60,669.9	764,488.4	86,029.4	911,187.7			
		1000 m buffer	210,338.7	2,170,049.9	450,919.1	2,831,307.7			
H	Kinuta Park	Within the park	318,804.5	22,232.7	102,434.1	443,471.3	3	1089	106
		500 m buffer	205,695.8	1,353,631.8	125,623.4	1,684,951.0			
		1000 m buffer	254,388.9	3,859,606.5	253,573.0	4,367,568.4			

*Note:* Bold indicates the largest areas among the parks, and italics indicate the smallest areas among the parks.

All these works were conducted using Geographic Information System (GIS) software, QGIS version 3.16.12 (https://www.qgis.org/en/site/, last accessed on April 19, 2024).

### 2.3 Camera Trap Survey

The camera trapping system, a complement of three to four cameras (Ltl‐acorn6210MC and Ltl‐acorn6210WMC), was widely separated and tied to a tree 1–1.5 m above the ground for each urban park. Specifically, they were placed at least approximately 200 m apart in a straight line (Nanakuni Countries Ridge Green Space: minimum distance 314.0 m, maximum distance 677.6 m, average distance 474.3 m; Nagayama Park: minimum distance 176.9 m, maximum distance 440.6 m, average distance 308.8 m; Hirayama Joshi Park: minimum distance 214.6 m, maximum distance 866.4 m, average distance 578.8 m; Nagaike Park: minimum distance 136.8 m, maximum distance 301.2 m, average distance 207.5 m; Oyamada Green Space: minimum distance 214.7 m, maximum distance 256.2 m, average distance 235.5 m; Sakuragaoka Park: Minimum distance: 194.0 m, maximum distance: 342.7 m, average distance: 287.4 m, Okamoto Seikado Green Space: Minimum distance: 102.9 m, maximum distance: 117.1 m, average distance: 114.9 m, Kinuta Park: Minimum distance: 195.9 m, maximum distance: 412.5 m, average distance: 247.3 m). Cameras were placed at each site within the forest interior along animal trails characterized by sparse undergrowth and compacted ground due to frequent use by wildlife at the eight parks. The observational span encompassed 365 consecutive days, starting on October 1, 2020, and concluding on September 30, 2021. The camera settings were configured to capture 15‐sequences, with a 30‐s interval between consecutive captures. Each camera underwent routine inspections approximately once a month, during which the batteries were replaced and the data were retrieved. Following Fukuda et al. (2008), we defined the camera duration as the period spanning from installation to the final verified image capture because camera operation stopped due to factors such as battery and memory depletion and camera malfunction.

Raccoon dog identification was based on the captured imagery; however, this potentially leads to the duplication of individuals (Tsukada et al. 2006; Shimada 2010). To avoid duplicate counts, we consolidated multiple consecutive captures of the same species within a 30‐min interval into a singular occurrence (Tsukada et al. 2006; Matsubayashi et al. 2009). We categorized the activity time of the raccoon dogs into four types according to Ikeda et al. (2016): daytime, nighttime, crepuscular, and cathemeral. We defined the crepuscular period as an interval of approximately 1 h post‐sunrise and sunset, according to Ota et al. (2019). The remaining temporal segments were apportioned into daytime (7:00–16:59), nighttime (19:00–4:59), and cathemeral (5:00–6:59 and 17:00–18:59). Because the season could affect the availability of food resources for raccoon dogs (as detailed below: Feeding habits analysis), we used four seasonal periods: spring (April and May, the average temperature at the Hachioji Observatory, which is the closest station to the surveyed parks, ranges from 13.3°C to 18.1°C, https://www.data.jma.go.jp/stats/etrn/view/nml_amd_ym.php?prec_no=44&block_no=0366&year=&month=&day=&view=p1, accessed on 11, July, 2025), numerous plants germinated and insects initiated activity; summer (June through September, 21.4°C–22.6°C), foliage expansion, and heightened insect activity; autumn (October and November, 21.5°C–16.4°C), encompassing the proliferation of many fruits; and winter (December through March, 5.9°C–8.0°C) reduced abundance of fruit and insects.

The aggregate duration of camera operation within each urban park was as follows: Nanakuni Ridge Green Space, 1037 days (four cameras); Naganuma Park, 931 days (three cameras); Hirayama Joshi Park, 1046 days (three cameras); Nagaike Park, 906 days (three cameras); Oyamada Green Space, 940 days (three cameras); Sakuragaoka Park, 1187 days (four cameras); Okamoto Seikado Green Space, 840 days (three cameras); and Kinuta Park, 1246 days (four cameras). The cumulative total was 8133 days.

### 2.4 Feeding Habits Analysis

Since raccoon dogs habitually defecate in certain places (Saeki 2008), fecal analysis was adopted in this study as it allows for efficient collection. From September 2020 to October 2021, we visited each park approximately once a month. We made periodic rounds to thoroughly explore the walkable routes to check two to four raccoon dog‐dropping sites, collecting a total of 155 feces. Among the urban parks surveyed, we were unable to collect and analyze feces from Naganuma Park because we could not confirm any feces sites there. Fecal surveys were conducted in the morning whenever possible to avoid decomposer activities such as beetle and flies. The number of fecal samples collected from each city park was as follows: Nanakuni Ridge Green Space (22), Hirayama Joshi Park (23), Nagaike Park (25), Oyamada Green Space (18), Sakuragaoka Park (32), Okamoto Seikado Green Space (14), and Kinuta Park (21). The samples were frozen in zippered plastic bags and thawed as required for the analysis.

Fecal contents were analyzed using the point frame method (Stewart 1967), which involves assessing the projected area of fecal contents by quantifying grid intersections following washing with a 0.5 mm interval sieve. This method expedites quantitative assessment compared to techniques that rely on weight or volume measurements (Sato et al. 2000; Takatsuki and Tatewaki 2012). Grid scores were recorded no fewer than 200 times per sample, as established by prior work (Takatsuki and Tatewaki 2012; Takatsuki 2013).

The washed fecal samples were classified into five categories: plant matter, animal matter, artificial matter, other matter, and unknown matter. Artificial matter, which constitutes a direct nutrient, is not often detected in fecal analyses because of digestion. Conversely, plastic and rubber bands used in food packaging consumed alongside residual food and raw garbage (Yamamoto and Kinoshita 1994) were detected in fecal analyses. Therefore, those judged utilizing artificial substances are used as food resources (Sasaki and Osawa 2022). Therefore, artificial substances in feces are likely to originate from residual food. Based on the outcomes, we determined the proportion of each category and assessed the presence of artificial matter in the feces per sample.

### 2.5 Statistical Analysis

Seasonal raccoon dog daytime activity for each urban park was determined by generalized linear models (GLMs) using binomial distributions (logit link) and the Wald test. As an explanatory variable, we used the urbanization area within the park, around the park within a 500 m buffer, or around the park within a 1000 m buffer. The park area was used as the offset term to regulate the effects of total habitat size. All areas were log‐transformed. Scabies infection may increase raccoon dog daytime activity (Sasaki and Osawa 2022). Therefore, based on a visual assessment of the videos, we ignored individuals identified as possibly infected with scabies.

The occurrence of artificial matter in the fecal samples from each urban park for each season was determined by the GLMs using a binomial distribution (logit link) and the Wald test using the same explanatory variable as the previous analysis. This analysis was performed for each season: autumn, 50 fecal samples; winter, 34 fecal samples; spring, 30 fecal samples; and summer, 41 fecal samples.

## 3 Results

### 3.1 Results of Camera Traps

Raccoon dogs were observed across all eight parks where the cameras were deployed, resulting in 156 sightings in Nanakuni Ridge Green Space, 564 in Naganuma Park, 610 in Hirayama Joshi Park, 231 in Nagaike Park, 297 in Oyamada Green Space, 809 in Sakuragaoka Park, 338 in Okamoto Seikado Green Space, and 1089 in Kinuta Park (Table 1). In addition to raccoon dogs, various other mammals, including raccoons ( *Procyon lotor* ), badgers (*Meles meles anakuma*), foxes ( *Vulpes vulpes japonica* ), palm civets ( *Paguma larvata* ), Japanese macaques ( *Macaca fuscata* ), Japanese hares ( *Lepus brachyurus* ), and rodents (*Rodentia*), were captured. These cameras also captured avian and reptilian species.

Raccoons, which are possibly competitors of raccoon dogs (Abe et al. 2006; Kuriyama et al. 2018; Osaki et al. 2019), were observed year‐round in all parks, except Sakuragaoka Park (detected only once with young raccoons). Raccoons are predominantly nocturnal (Lotze and Anderson 1979) and their presence or absence can influence the activity of raccoon dogs. Therefore, we did not include data from Sakuragaoka Park in the analysis.

### 3.2 Relationship Between the Daytime Activity of Raccoon Dogs and Urbanization

The GLM analyses found that, in autumn, raccoon dog daytime activity was negatively correlated with the urban area within the parks (0 m buffer). In contrast, it was positively correlated with the urban area within the 1000 m buffers at the 5% significance level (Table 2, bold with large font). The urban areas within the 500 m buffers were not estimated as a parameter of the GLMs because the urban area, which was used as an explanatory variable, was dichotomous depending on the occurrence of daytime activity. In winter, there was no correlation between daytime activity and all types of urban areas (Table 2). In spring, urban areas within both the 500 and 1000 m buffers were positively correlated with the occurrence of daytime activity at the 10% significance level (Table 2, bold with large font). In summer, the urban areas within the parks (0 m buffer) were positively correlated at the 10% significance level. In contrast, the urban areas within the 500 and 1000 m buffers had no significant correlation with daytime activity at the 10% significance level (Table 2).

**TABLE 2:** GLM (generalized linear model) results for the relationship between the daytime activity of raccoon dogs and areas of urbanization in each park.

Season	Buffer size (m)	Estimate	Std. error	*z*	*p*
Autumn	0	−2.047	0.769	−2.661	0.008**
	(Intercept)	7.015	7.131	0.984	0.325
	500				
	(Intercept)				
	1000	5.555	2.490	2.231	0.026*
	(Intercept)	−91.757	35.811	−2.562	0.010
Winter	0				
	(Intercept)				
	500	−0.455	1.936	−0.235	0.814
	(Intercept)	−4.237	26.233	−0.162	0.872
	1000	−0.687	2.017	−0.341	0.733
	(Intercept)	−0.3834	29.436	−0.013	0.990
Spring	0	−0.496	0.653	−1.207	0.448
	(Intercept)	−7.155	5.929	−0.759	0.227
	500	3.331	1.892	1.760	**0.078**.
	(Intercept)	−56.376	25.329	−2.226	0.026
	1000	4.157	2.192	1.896	**0.058**.
	(Intercept)	−71.650	31.533	−2.272	0.023
Summer	0	2.037	1.105	1.844	**0.065**.
	(Intercept)	−30.826	10.032	−3.073	0.002
	500	2.319	1.494	1.522	0.121
	(Intercept)	−43.592	20.138	−2.165	0.030
	1000	2.425	1.588	1.527	0.127
	(Intercept)	−47.47	22.993	−2.065	0.039

*Note:* For the autumn 500 m buffer and the winter within the parks, the logistic regression model parameters could not be estimated because the urban landscapes used as an explanatory variable were dichotomous; it depended on whether raccoons were active during the day. The *p* values indicate the results of the Wald test.

< 0 “***” < 0.001 “**” < 0.01 “*” < 0.05 “.” < 0.1 “ ” < 1.

As shown by the GLM analysis, daytime activities of raccoon dogs varied greatly depending on the urban park and season (Figure S1).

### 3.3 Contents of Raccoon Dog Feces

Table 3 summarizes the occupancy rates for each season according to the four fecal content categories: plant matter, animal matter, artificial matter, and unknown matter. For artificial matter, discernible items included plastic fragments, rubber bands, aluminum foil, fabric, silver paper, plastic bags, and various types of food packaging. Across all the parks, the occupancy rate of plant matter, including seeds, was dominant during autumn. In winter, the occupancy rate of plant matter including seeds, decreased, and the occupancy rate of artificial matter increased rapidly. In spring, the occupancy rate of animal matter was greater compared to that in winter. In summer, the occupancy rate of plant matter surged, becoming equivalent to that of animal matter. Of the seven urban parks, Kinuta Park and Okamoto Seikado Green Space had the highest occupancy rates for artificial matter for all seasons. In comparison, the artificial matter occupancy rate in the remaining six parks only increased during winter and spring.

**TABLE 3:** Contents (%) of raccoon dog feces collected from each urban park and analyzed using the point frame method.

Season	Park	Natural matter		Artificial matter	Others matter	Unknown matter
		Plant matter	Animal matter			
Autumn	Nanakuni Ridge Green Space	75.6	19.0	0.1	4.2	1.1
	Naganuma Park	—	—	—	—	—
	Hirayama Joshi Park	80.1	13.3	1.8	3.6	1.2
	Nagaike Park	86.0	8.8	0.7	4.6	0.0
	Oyamada Green Space	72.4	24.4	0.0	3.2	0.0
	Sakuragaoka Park	82.8	13.3	0.0	3.2	0.7
	Okamoto Seikado Green Space	73.0	10.0	13.3	3.8	0.0
	Kinuta Park	67.9	14.4	11.2	6.4	0.1
Winter	Nanakuni Ridge Green Space	65.9	25.4	1.4	6.5	0.8
	Naganuma Park	—	—	—	—	—
	Hirayama Joshi Park	51.4	22.6	17.8	6.8	1.5
	Nagaike Park	49.1	35.9	11.1	3.4	0.6
	Oyamada Green Space	67.2	23.0	5.7	3.3	0.8
	Sakuragaoka Park	63.1	20.0	9.1	6.1	1.7
	Okamoto Seikado Green Space	43.4	22.6	26.0	7.8	0.2
	Kinuta Park	48.9	28.2	19.2	3.7	0.0
Spring	Nanakuni Ridge Green Space	37.5	57.4	0.3	3.6	1.2
	Naganuma Park	—	—	—	—	—
	Hirayama Joshi Park	30.8	51.6	11.7	2.1	3.8
	Nagaike Park	27.4	69.5	0.0	1.9	1.2
	Oyamada Green Space	35.7	59.9	1.7	2.4	0.2
	Sakuragaoka Park	34.8	59.9	1.3	2.5	1.5
	Okamoto Seikado Green Space	25.5	56.2	16.1	2.2	0.0
	Kinuta Park	24.7	31.6	21.9	21.3	0.5
Summer	Nanakuni Ridge Green Space	52.5	42.1	0.0	3.5	1.9
	Naganuma Park	—	—	—	—	—
	Hirayama Joshi Park	51.5	42.6	2.3	2.3	1.2
	Nagaike Park	51.2	42.1	0.0	5.2	1.5
	Oyamada Green Space	61.0	37.0	0.0	2.0	0.0
	Sakuragaoka Park	50.3	42.1	0.8	5.7	1.0
	Okamoto Seikado Green Space	50.1	37.7	9.0	3.3	0.0
	Kinuta Park	41.9	36.1	17.2	4.5	0.2

### 3.4 Relationship Between the Occurrence of Artificial Matter and Urbanization

The GLM analyses found that in autumn, the occurrence of artificial matter was positively correlated with urbanized areas within both the 500 and 1000 m buffers at the 5% significance level (Table 4, bold with large font). In winter, there was no correlation between the occurrence of artificial matter and urbanized areas (Table 4). However, in both spring and summer, urbanized areas within both the 500 and 1000 m buffers were positively correlated with artificial matter at the 5% significance level (Table 4, bold with large font).

**TABLE 4:** GLM results for the relationship between the use of artificial matter as a food resource and urbanization.

Season	Buffer size (m)	Estimate	Std. error	*z*	*p*
Autumn	0	7.42 × 10^−5^	5.82 × 10^−5^	1.275	0.202
	(Intercept)	−1.895	0.816	−2.323	0.020
	500	2.18 × 10^−6^	1.01 × 10^−6^	2.165	0.030*
	(Intercept)	−2.887	0.991	−2.914	0.004
	1000	1.51 × 10^−6^	4.95 × 10^−7^	3.052	0.002**
	(Intercept)	−4.367	1.182	−3.693	0.000
Winter	0	6.49 × 10^−5^	1.05 × 10^−4^	0.617	0.537
	(Intercept)	1.600	1.204	1.329	0.184
	500	9.09 × 10^−7^	1.95 × 10^−6^	0.466	0.641
	(Intercept)	1.544	1.688	0.926	0.354
	1000	8.5 × 10^−7^	1.04 × 10^−6^	0.816	0.414
	(Intercept)	0.538	2.087	0.258	0.797
Spring	0	−2.18 × 10^−5^	6.61 × 10^−5^	−0.329	0.742
	(Intercept)	0.260	0.871	0.299	0.765
	500	3.32 × 10^−6^	1.37 × 10^−6^	2.427	0.015*
	(Intercept)	−2.648	1.139	−2.325	0.020
	1000	1.71 × 10^−6^	7.98 × 10^−7^	2.142	0.032*
	(Intercept)	−3.447	1.595	−2.161	0.031
Summer	0	3.32 × 10^−6^	5.95 × 10^−5^	0.056	0.956
	(Intercept)	−0.692	0.721	−0.961	0.337
	500	2.82 × 10^−6^	1.23 × 10^−6^	2.293	0.022*
	(Intercept)	−3.166	1.189	−2.662	0.008
	1000	2.8 × 10^−6^	1.19 × 10^−6^	2.358	0.018*
	(Intercept)	−6.692	2.577	−2.597	0.009

*Note:* The *p* values indicate the results of the Wald test.

< 0 “***” < 0.001 “**” < 0.01 “*” < 0.05 “.” < 0.1 “ ” < 1.

## 4 Discussion

This study tested the relationships between raccoon dog activity time, feeding habits, and the degree of urbanization. The results suggested that while raccoon dogs remained basically nocturnal, they could shift their feeding habits to artificial food resources according to the degree of urbanization. Therefore, our hypothesis is partially supported. This insight suggests that there may be a link between raccoon dog activity time and the use of artificial food resources.

The results of the camera trap survey showed that raccoon dogs were present in all eight parks regardless of the size of the green space that served as their primary habitat or the environment surrounding the parks. This is consistent with previous studies (Sonoda and Kuramoto 2008; Saito and Koike 2013). Therefore, urban parks are becoming important habitats for raccoon dogs in the Tokyo area.

Previous studies have indicated that many urban wildlife change to full nocturnal or reduced daytime activity to avoid humans (Riley et al. 2003; Ditchkoff et al. 2006; Gaynor et al. 2018; Stark et al. 2020). Therefore, we predicted that raccoon dogs would become fully nocturnal according to the degree of urbanization to avoid humans. However, we found no such trend in this study, excluding parks in autumn. Instead, we found that both in autumn and spring, urbanized areas surrounding parks were positively correlated with daytime activity. In summer, urbanized areas within parks were positively correlated with daytime activity. These results suggest that raccoon dogs may approach rather than avoid humans. When wildlife change their ecology and behavior, such as changes in activity time, they often exhibit adaptations that confer an advantage within their habitat (Gaynor et al. 2018). Therefore, the raccoon dog activity time identified in this study may have a strategic advantage when they are inhabiting an urban environment.

The results of the fecal survey showed that raccoon dogs used artificial matter as a food resource in all eight parks. Given the identified artificial matter and the olfactory‐driven foraging behavior of medium‐sized mammals, including raccoon dogs (Herrera 1989; Takatsuki 2018), it is likely that the raccoon dogs were attracted to the scent of leftover food or ingested the artificial matter with leftover food.

There were apparent variations in the occupancy rates of the five categories (i.e., plant matter, animal matter, artificial matter, other matter, and unknown matter) in the raccoon dog feces throughout the year, depending on the park and season. Both the Okamoto Seikado Green Space and Kinuta Park fecal samples tended to contain relatively large amounts of artificial matter. However, the Nagaike Park, Oyamada Green Space, and Sakuragaoka Park fecal samples tended to contain relatively small amounts of artificial matter. These results likely reflect the effect of urbanization on the feeding habits of raccoon dogs. Both the Okamoto Seikado Green Space and Kinuta Park are in Setagaya Ward, which is particularly urbanized. Thus, raccoon dogs inhabiting areas with significant urbanization tend to depend on artificial matter as their food resource. For all the parks, the occupancy rate of artificial matter tended to increase only in winter, which probably reflects a decrease in the availability of biological resources such as fruits and insects. Moreover, for the other seasons, especially autumn and summer, the occupancy rate of artificial matter tended to decrease for all the parks.

However, the relationship between urbanization and using artificial matter as a food resource was confirmed only outside the parks, regardless of season. A trend toward the presence of artificial matter was observed during fall, spring, and summer, especially as the area of anthropogenic landscapes outside the parks increased. This trend suggests that as the area of anthropogenic landscapes expands during fall, spring, and summer, raccoon dogs become more dependent on artificial matter as their food resource. As opportunistic omnivores, raccoon dogs adapt their foraging behavior according to resource availability (Ohdachi et al. 2015). Therefore, as urbanization increases the amount of artificial food resources within their range, their dependence on such sources may change. However, no such trend was observed in winter. This suggests that raccoon dogs compensate for the lack of natural food resources by actively using artificial food resources in winter regardless of their environment.

### 4.1 Relationship Between Activity Time and Resource Availability

In both autumn and spring, raccoon dogs increased their daytime activity according to the degree of surrounding urbanization, with a concomitant increase in the use of artificial food resources. These results suggest that raccoon dogs are more likely to use artificial food resources as food resources outside rather than inside the parks. One possible mechanism underlying this pattern is human activity, which may influence the availability of food resources for raccoon dogs. One of the potential influential factors is the timing of garbage disposal, which is typically scheduled during the day rather than at night. Previous studies have shown that raccoon dogs appear in urban areas in parallel with forested areas (Yamamoto 1993; Kaneko et al. 2008), and their foraging behavior includes regular visits to municipal garbage collection sites (Kaneko et al. 1998). Our results suggest that raccoon dogs may actively use easily accessible artificial food resources as a food resource. As anthropogenic landscapes outside the park expand, there is a commensurate increase in the number of locations that provide easily accessible artificial food resources in the daytime, exemplified by garbage dumps. Thus, daytime activities in places with heightened human presence may provide a strategic advantage.

However, in summer, daytime activity increased with the area of urbanization within the parks, and the use of artificial matter as a food resource increased with the increased area of urbanization outside the parks. Therefore, multiple factors might influence raccoon dog activity time and food resource use during the summer. First, the number of park users increases during summer due to leisure use, which may increase the amount of artificial matter in parks that could serve as a food resource. Second, since summer is the nursing season for raccoon dogs, they may be more wary of human activity than during other seasons and may avoid foraging outside the park or in other areas with high human activity. In addition, young raccoon dogs during the nursing season are vulnerable to nighttime predators and hypothermia, so raccoon dog adults may intentionally engage in daytime activities to protect their young (Zoller and Drygala 2013). Thus, the duration of summer daytime activity is likely to be modified by several factors, not just urbanization.

In winter, there was no correlation between the degree of urbanization and daytime activity or the use of artificial matter as a food resource. As winter is characterized by the reduced availability of natural resources, such as fruits and insects, artificial matter utilization was the highest for all parks and seasons. This result suggests that raccoon dogs in urban parks resort to artificial food resources to overcome the deficiency in naturally occurring food resources during winter, independent of activity time.

### 4.2 Limitation and Challenge

This study provides important insights into the behavioral changes in raccoon dogs within urbanized regions; however, some unresolved issues must be acknowledged. First, the study was conducted exclusively in urban parks, leaving out raccoon dogs in non‐urban areas. As raccoon dogs are known to occasionally exhibit daytime activity (Ohdachi et al. 2015), it would be of interest to explore the circumstances under which such behavior occurs in non‐urban settings. Second, individual identification was not performed in this study. Given that raccoon dogs generally have territories (Ohdachi et al. 2015), it is possible that the individuals detected by camera traps were biased. Nonetheless, because the analysis was based on data collected from multiple parks, we believe that the overall findings are robust and not significantly affected by this bias. Addressing these limitations is an important direction for future research.

## 5 Conclusions

We examined the adaptation of raccoon dogs to urban environments by focusing on their activity time and feeding habits. The results suggest that their activity time and feeding habits may be related to their adaptation to urban areas. As the area of their primary habitat increased (anthropogenic landscapes around urban parks), raccoon dogs were observed to be more active during the daytime and to use artificial food resources. This is thought to be due to daytime activity and the use of artificial food resources, which provide an advantage for urban adaptation, depending on the supply time of artificial food resources and other factors. Thus, with increasing urbanization, feeding habits and activity time may change. Activity patterns and the ability to flexibly adapt feeding habits are critical factors for wildlife to thrive in urbanized areas (Ditchkoff et al. 2006). Our study suggests that activity time and feeding habits are interrelated and should be discussed in combination. Further research is required because the effects observed in our study may also occur in other wildlife in urban habitats.

## Author Contributions

**Shoya Sasaki:** conceptualization (lead), data curation (lead), formal analysis (equal), investigation (lead), methodology (equal), project administration (lead), resources (lead), software (lead), visualization (lead), writing – original draft (lead), writing – review and editing (equal). **Takeshi Osawa:** conceptualization (supporting), data curation (supporting), formal analysis (supporting), funding acquisition (lead), investigation (supporting), methodology (equal), project administration (equal), resources (equal), software (equal), supervision (lead), validation (lead), visualization (equal), writing – original draft (supporting), writing – review and editing (lead).

## Conflicts of Interest

The authors declare no conflicts of interest.

## Supporting information


**Figure S1:** Estimation of the diurnal activity of raccoon dogs in each camera by season and survey site using kernel density estimation.

## Data Availability

All data set and analyzed code were attached as Supporting Information.
