# Supplementary material for: Influence of Food Resource Availability on the Activity Time of Raccoon Dogs (Nyctereutes procyonoides) in Urban Parks in Tokyo
Source: Ecol Evol. 2025 Aug 8;15(8):e71966. doi: 10.1002/ece3.71966 (PMC12332538; doi:10.1002/ece3.71966)

**Supplemental Information for:**

Supplementary Figure 1. Estimation of the diurnal activity of raccoon dogs in each camera by season and survey site using kernel density estimation.

A：Nanakuni Ridge Green Space，B：Naganuma Park，C：Hirayama Joshi Park，D：Nagaike Park，E：Oyamada Green Space，F：Sakuragaoka Park，G：Okamoto Seikado Green Space，H：Kinuta Park

The times at which raccoon dogs were detected by each camera trap were aggregated by season, and the probability of occurrence was estimated for each time of day. The estimation was conducted using the overlap package in R.

**Autumn**


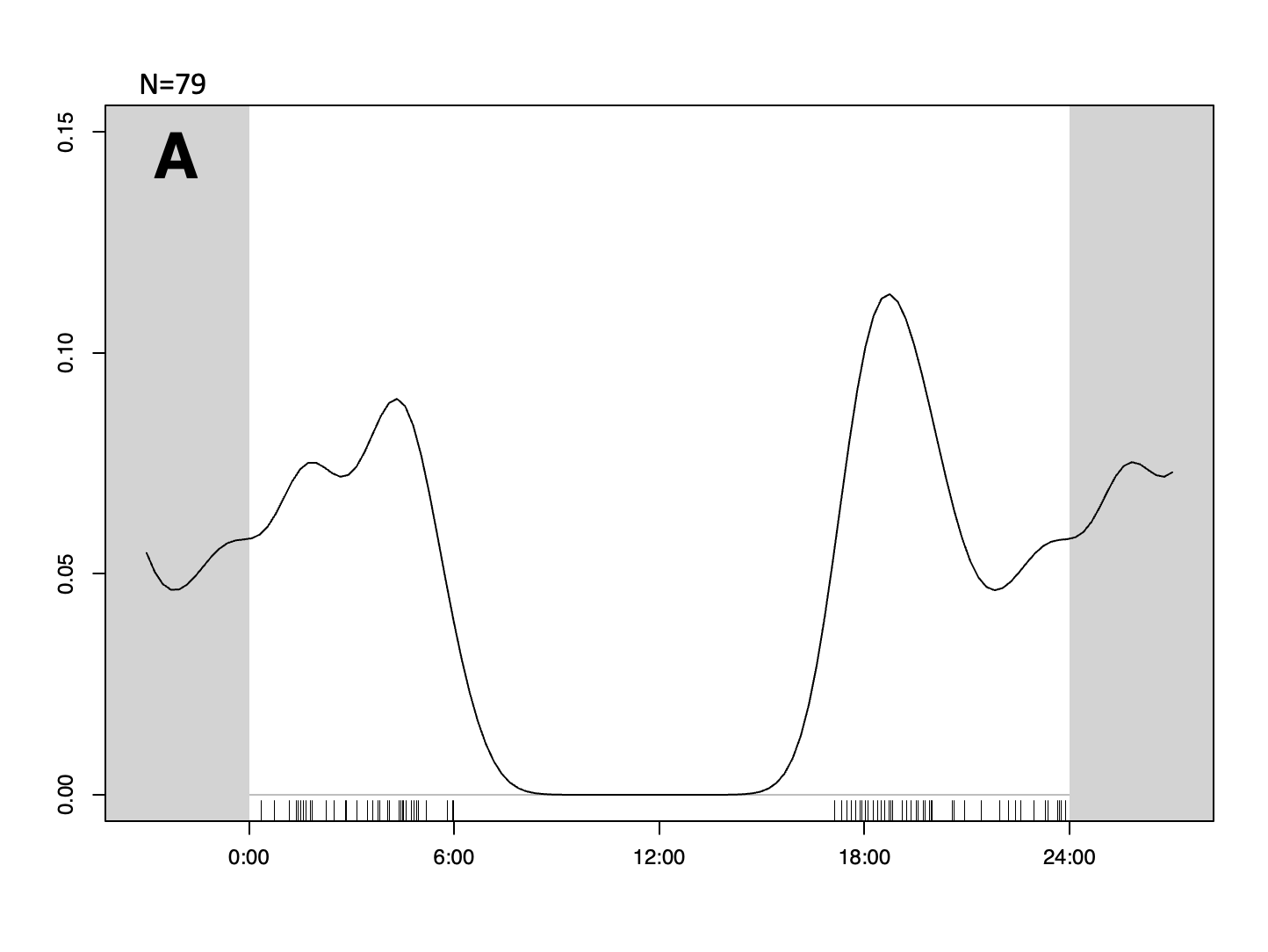

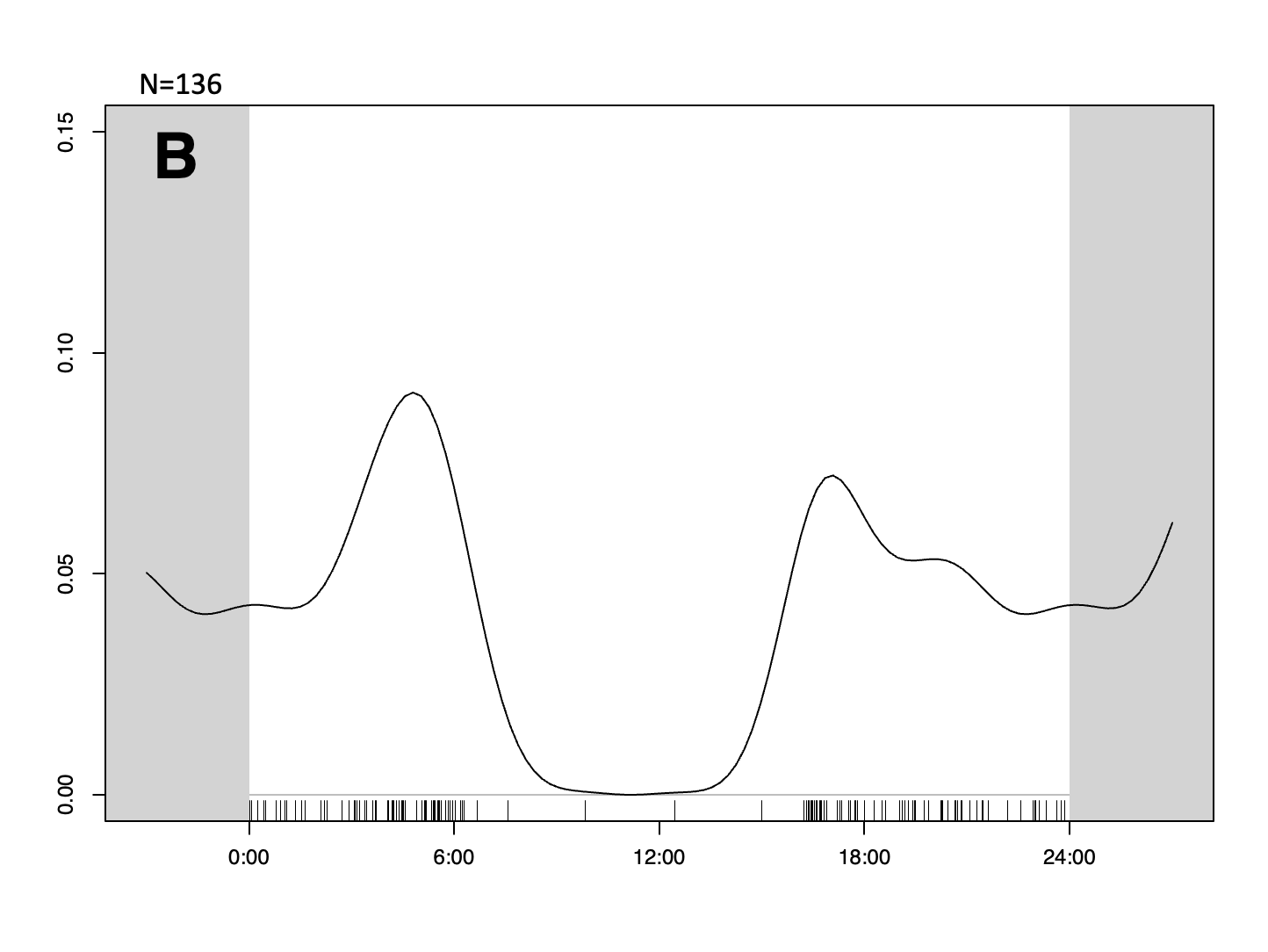

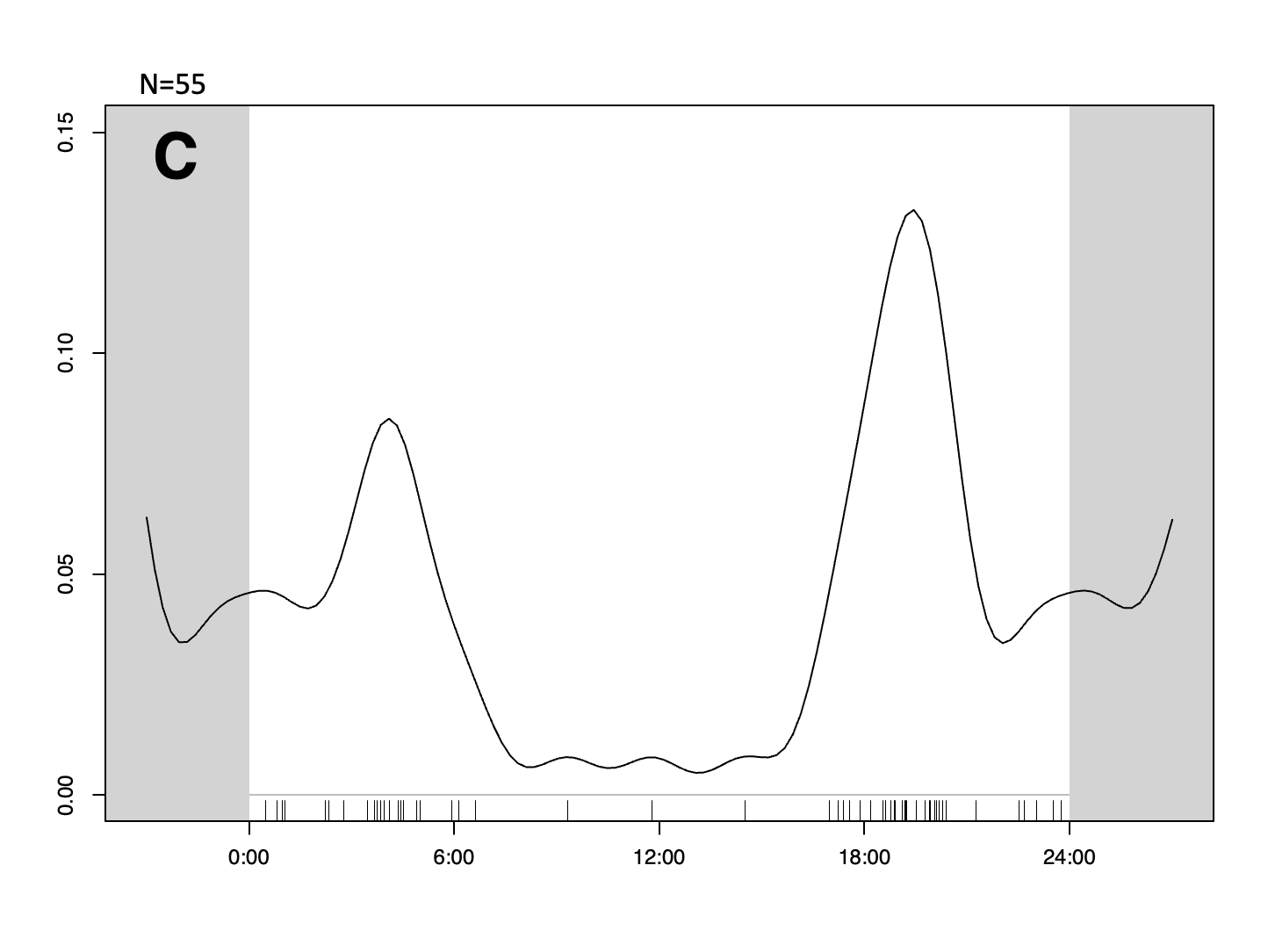

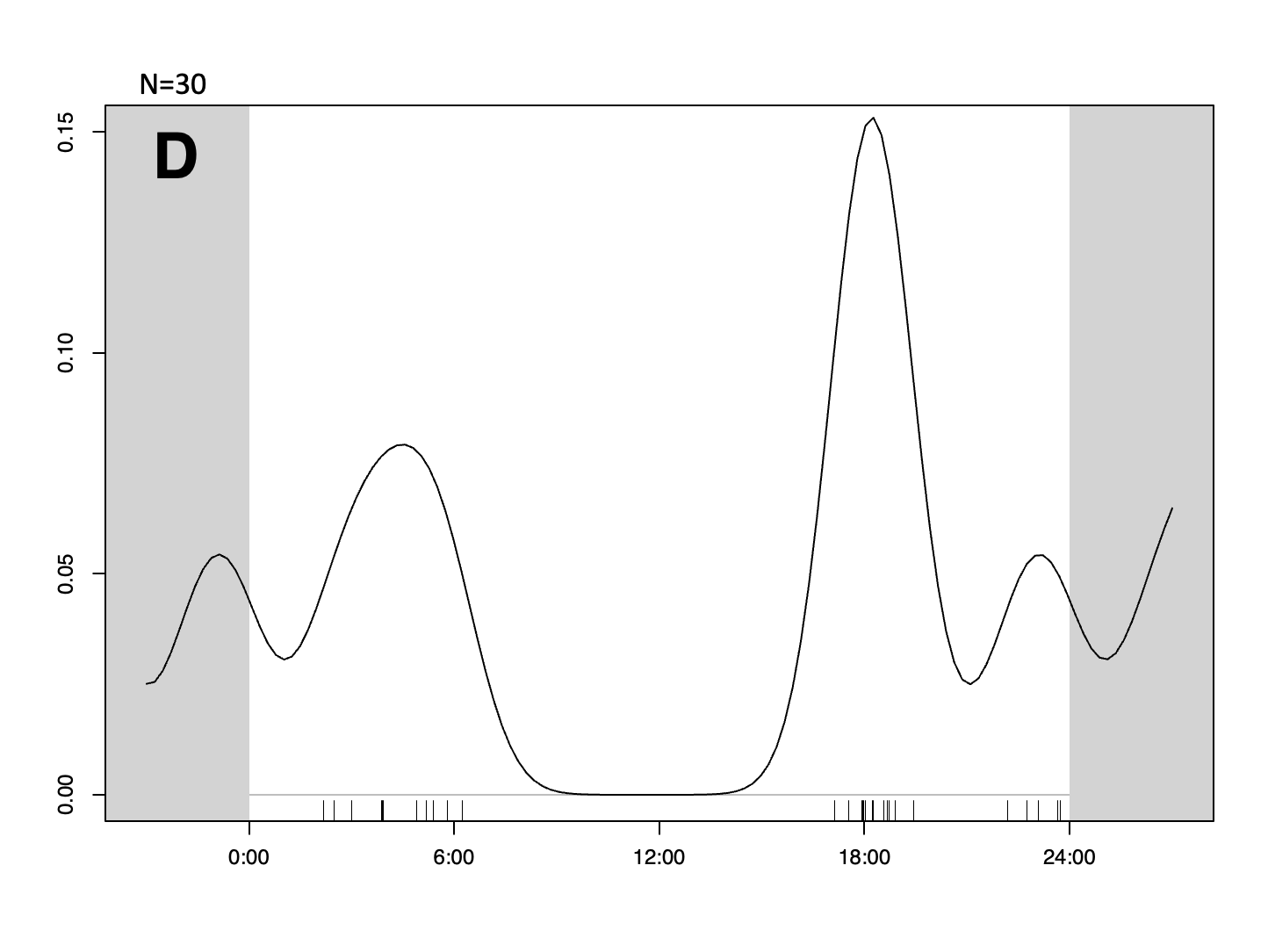

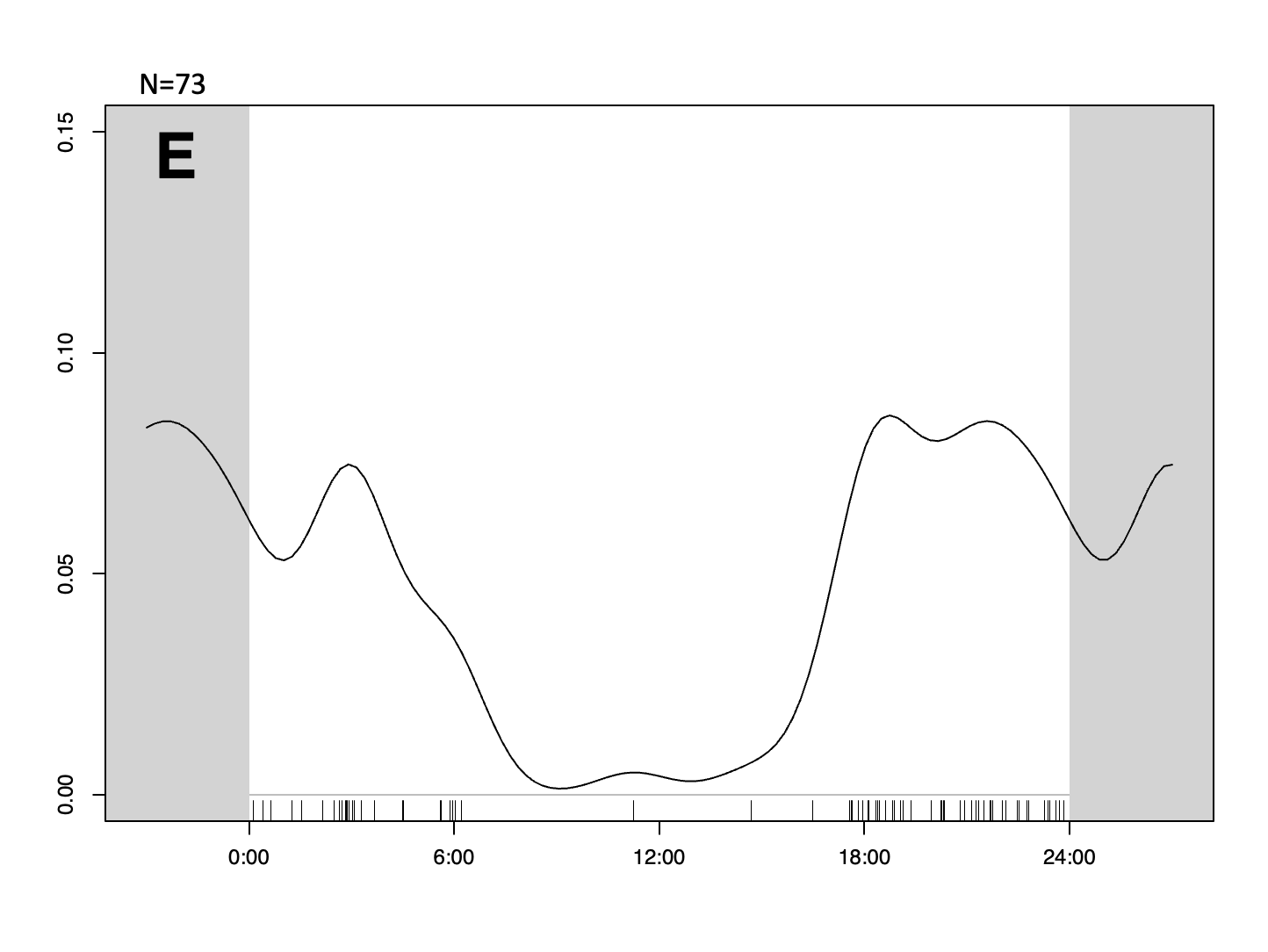

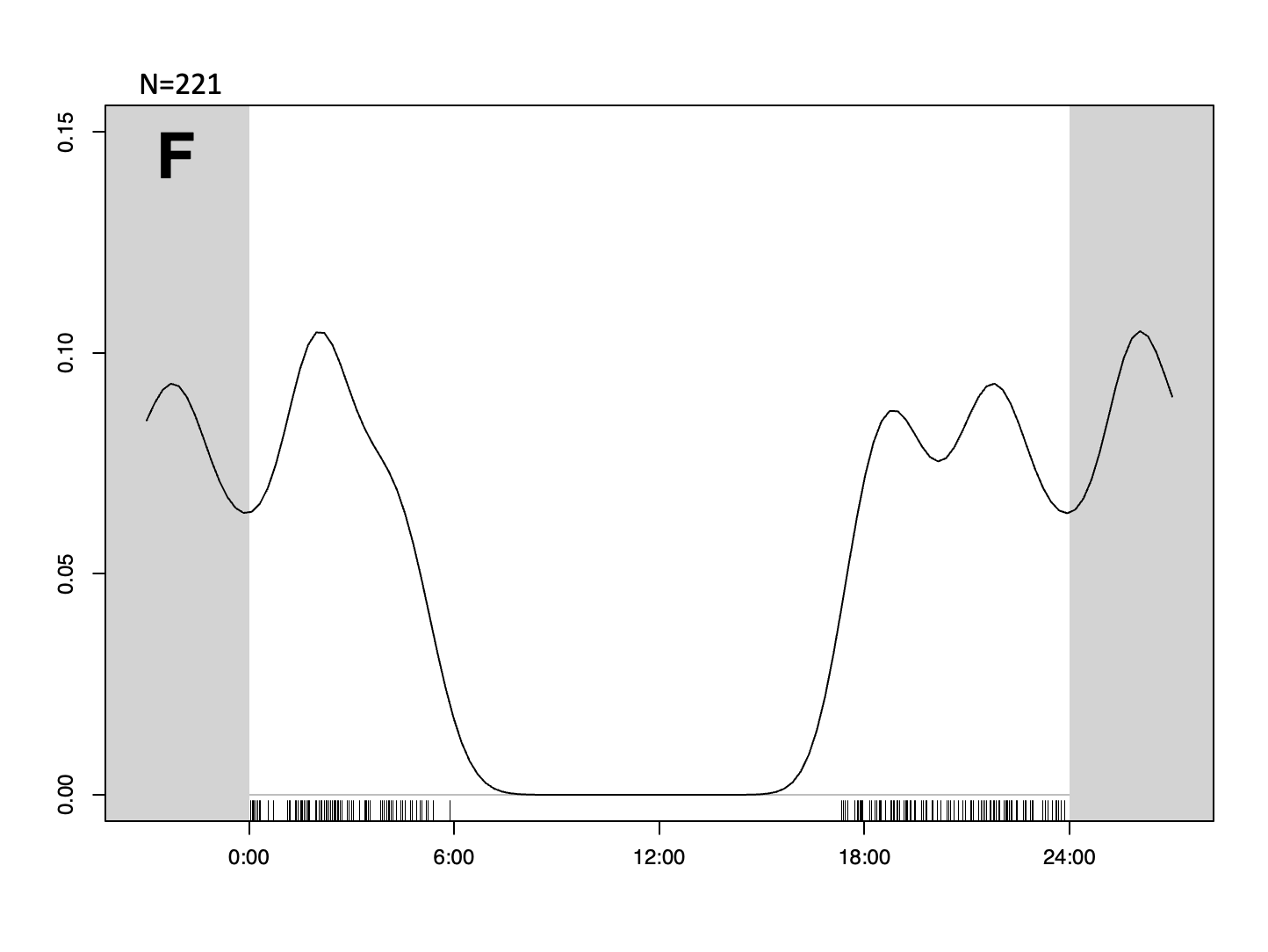

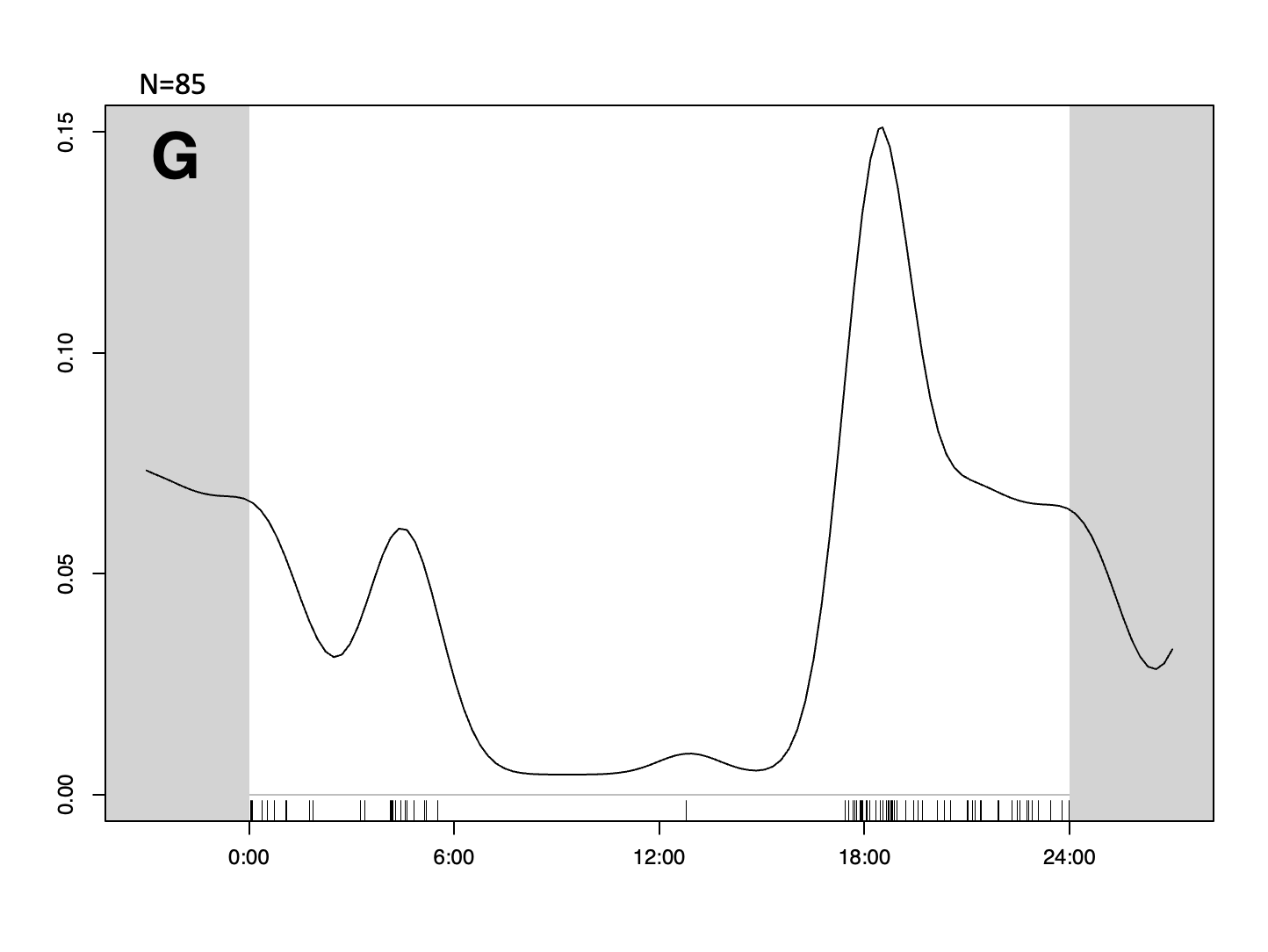

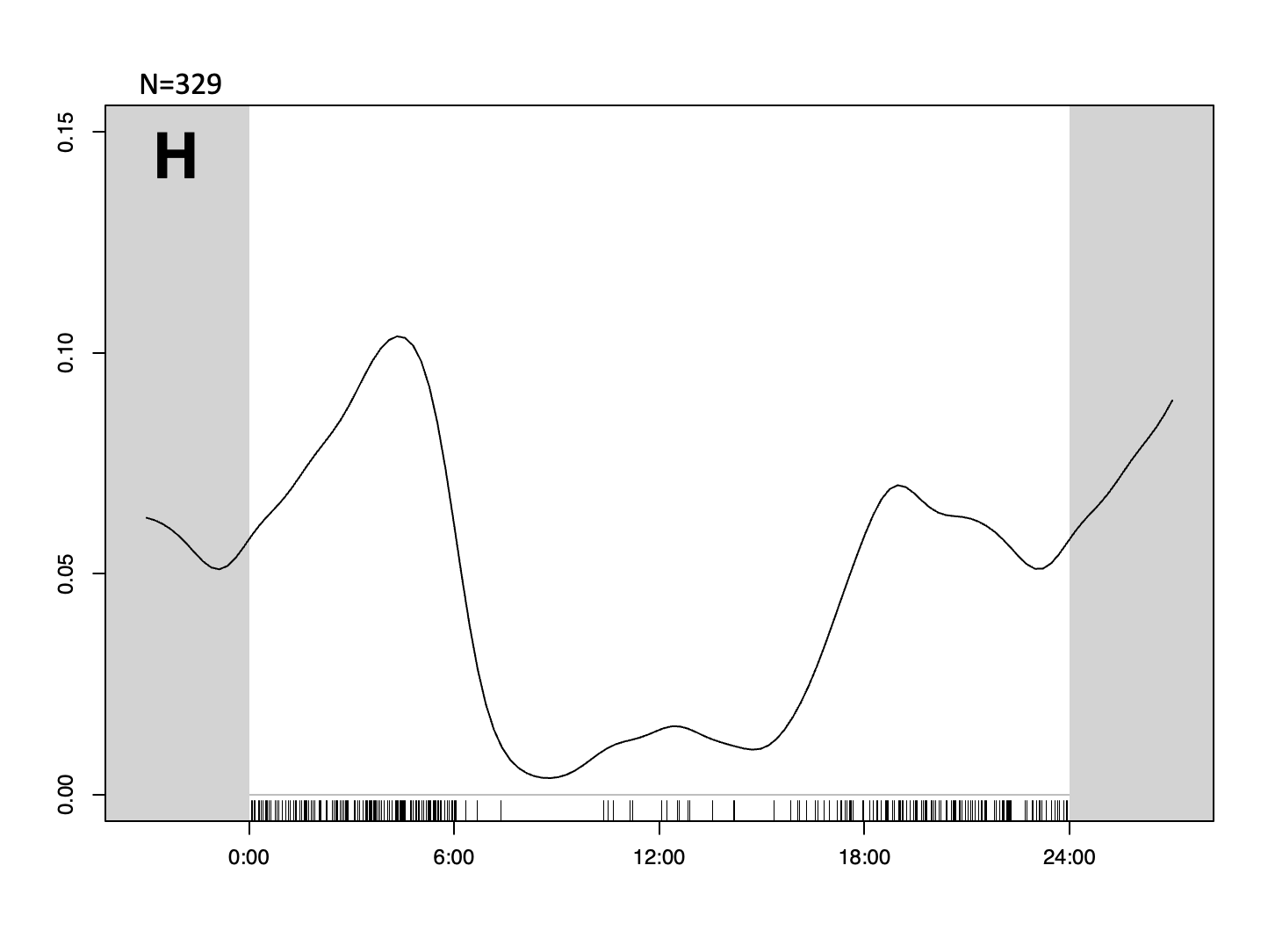


**Winter**


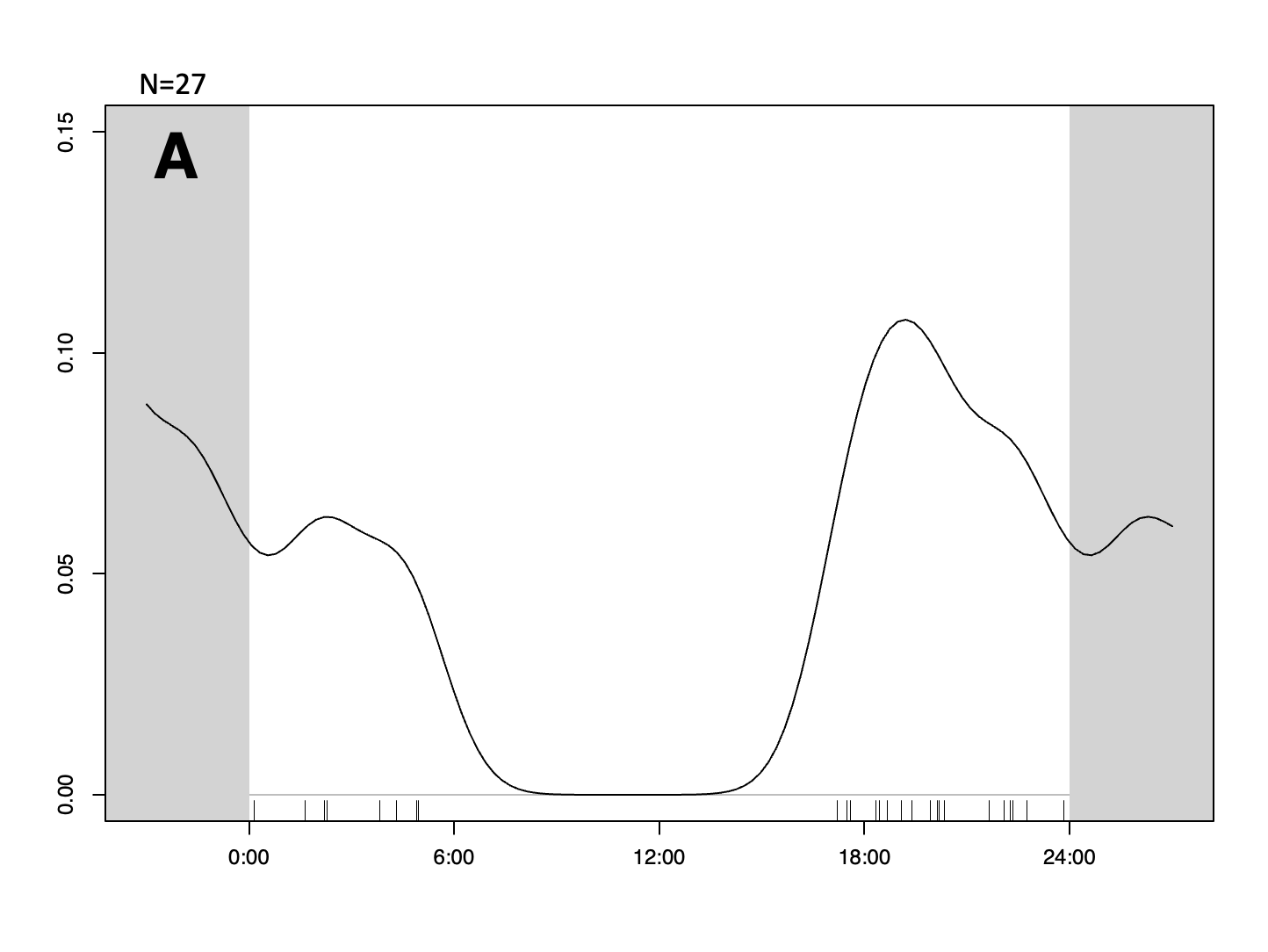

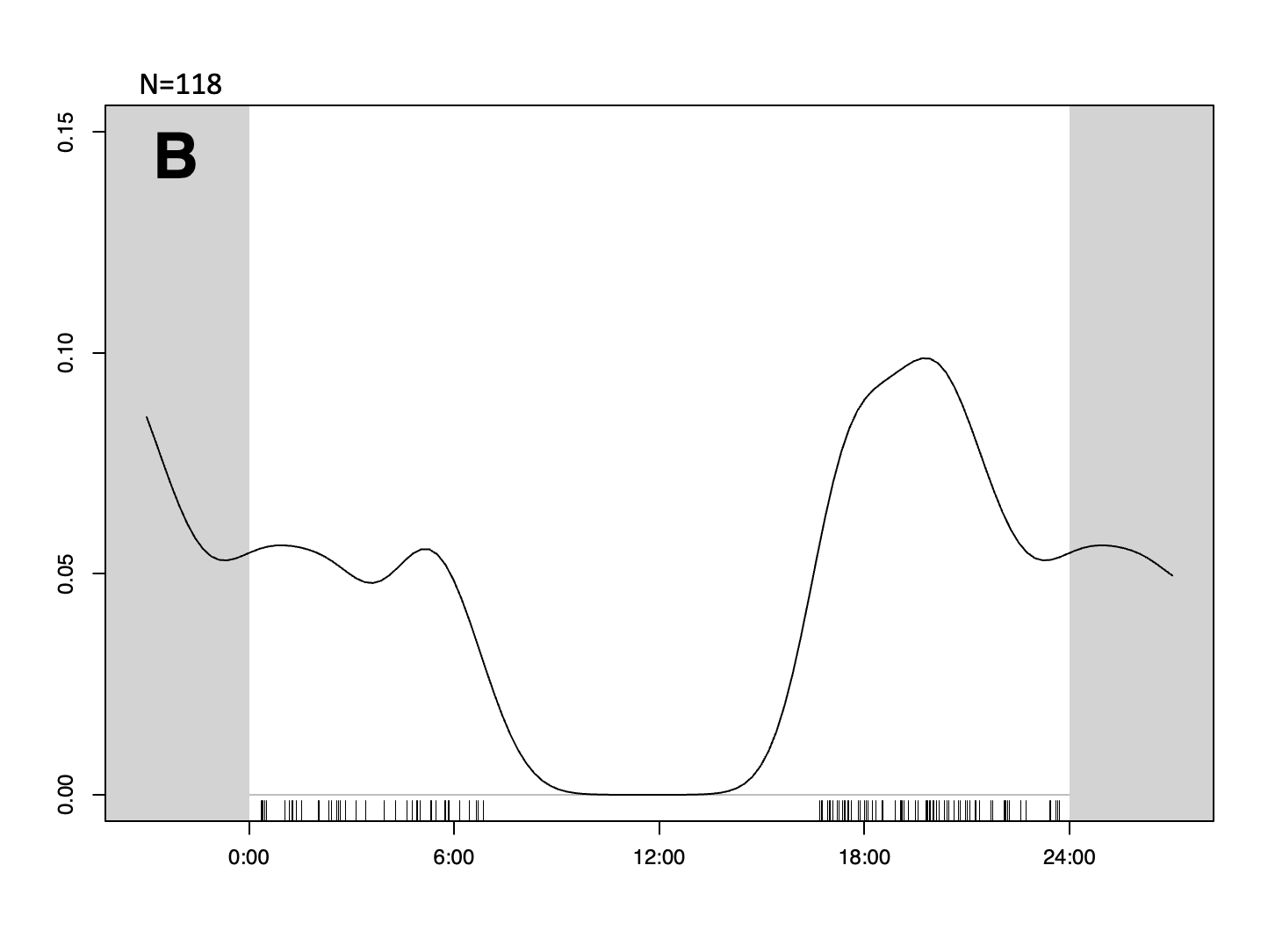

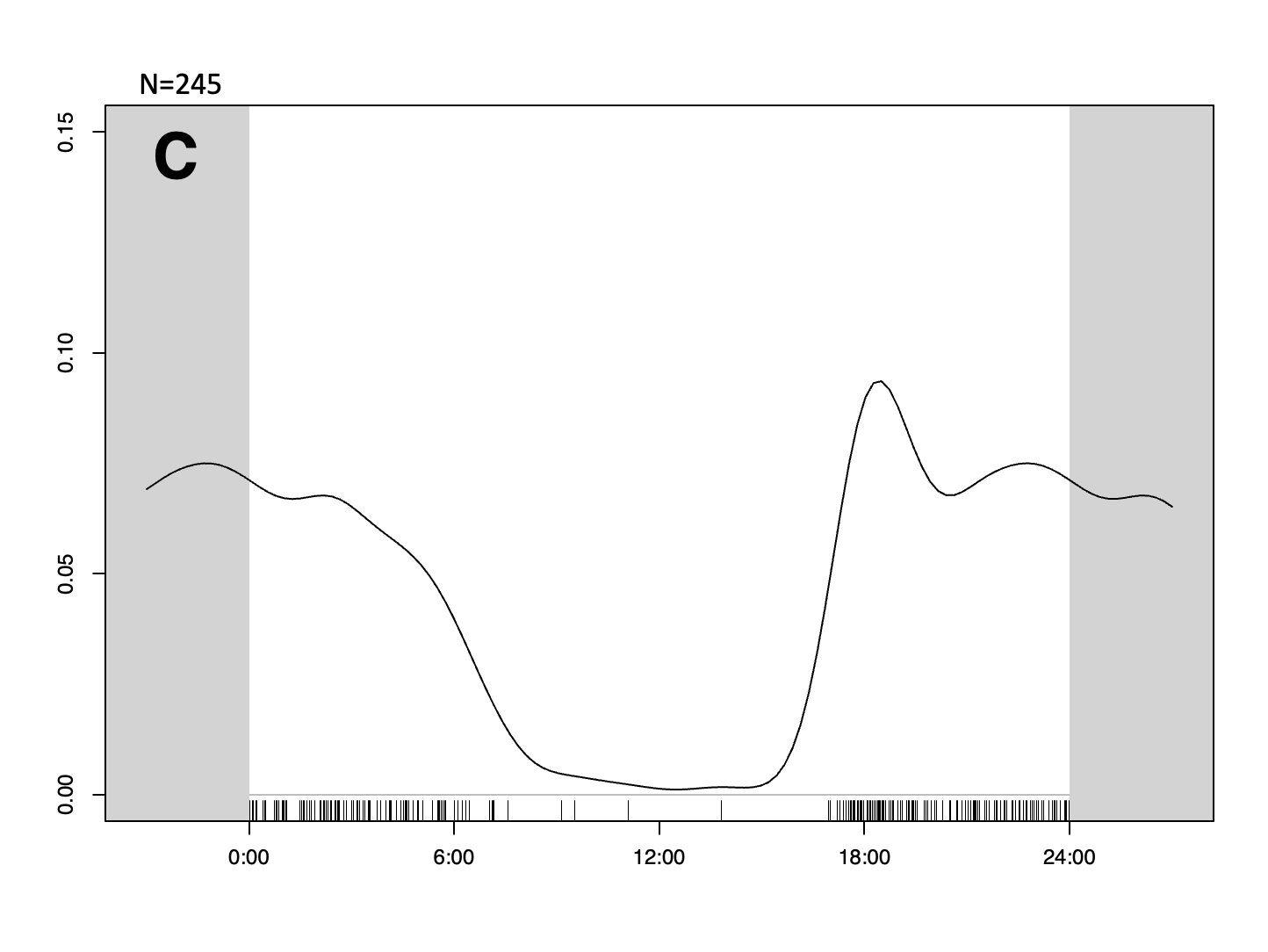

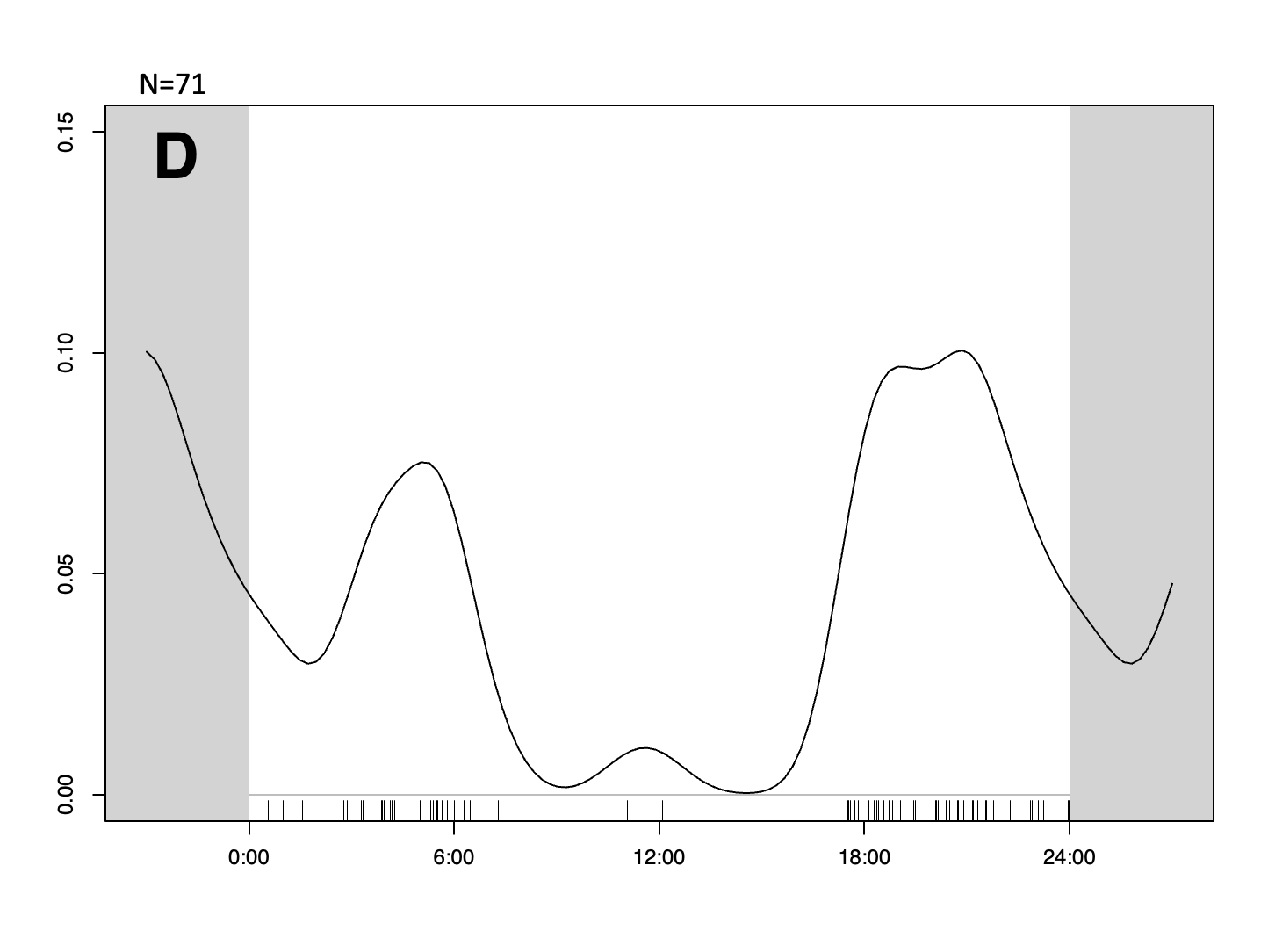

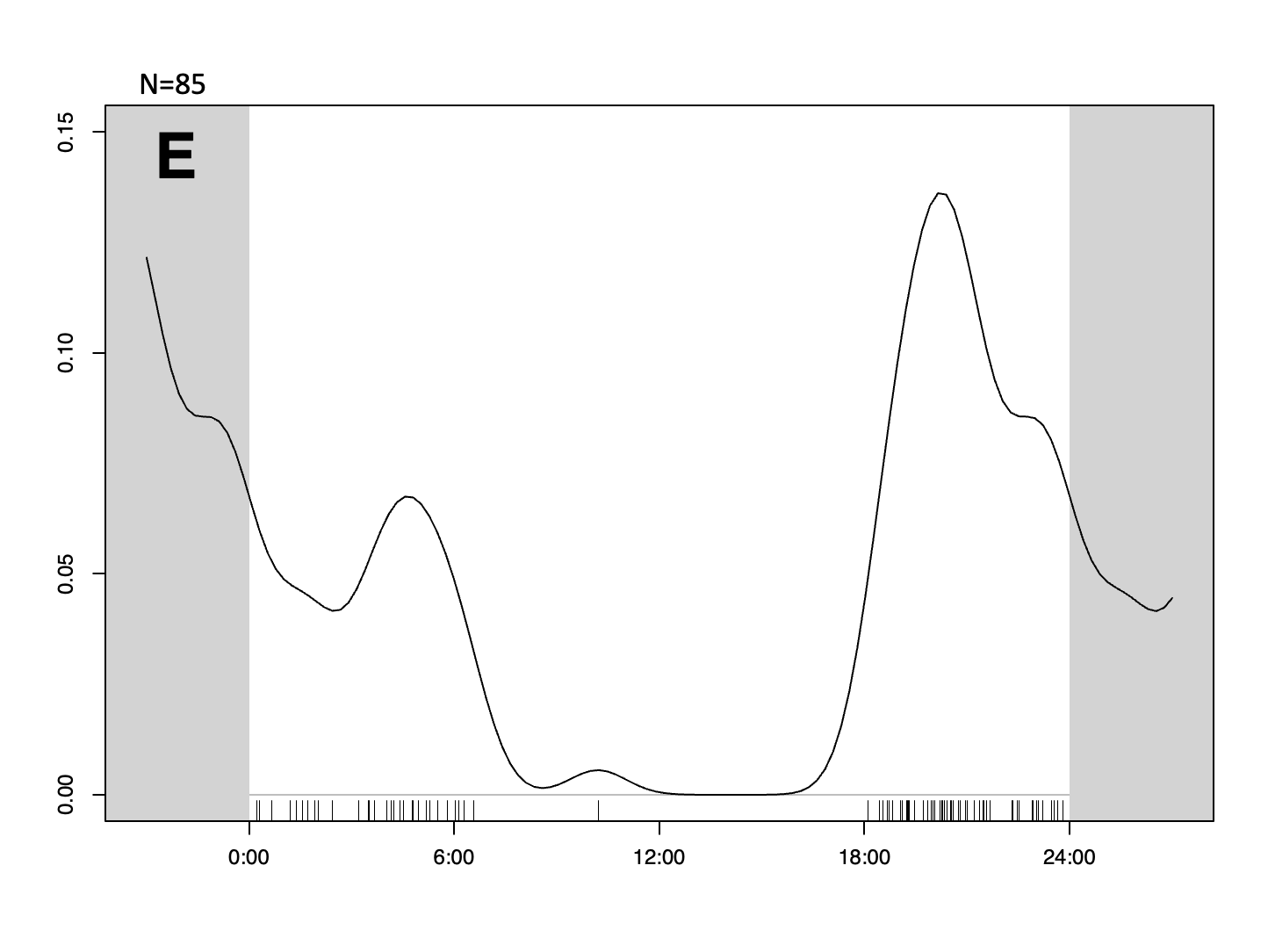

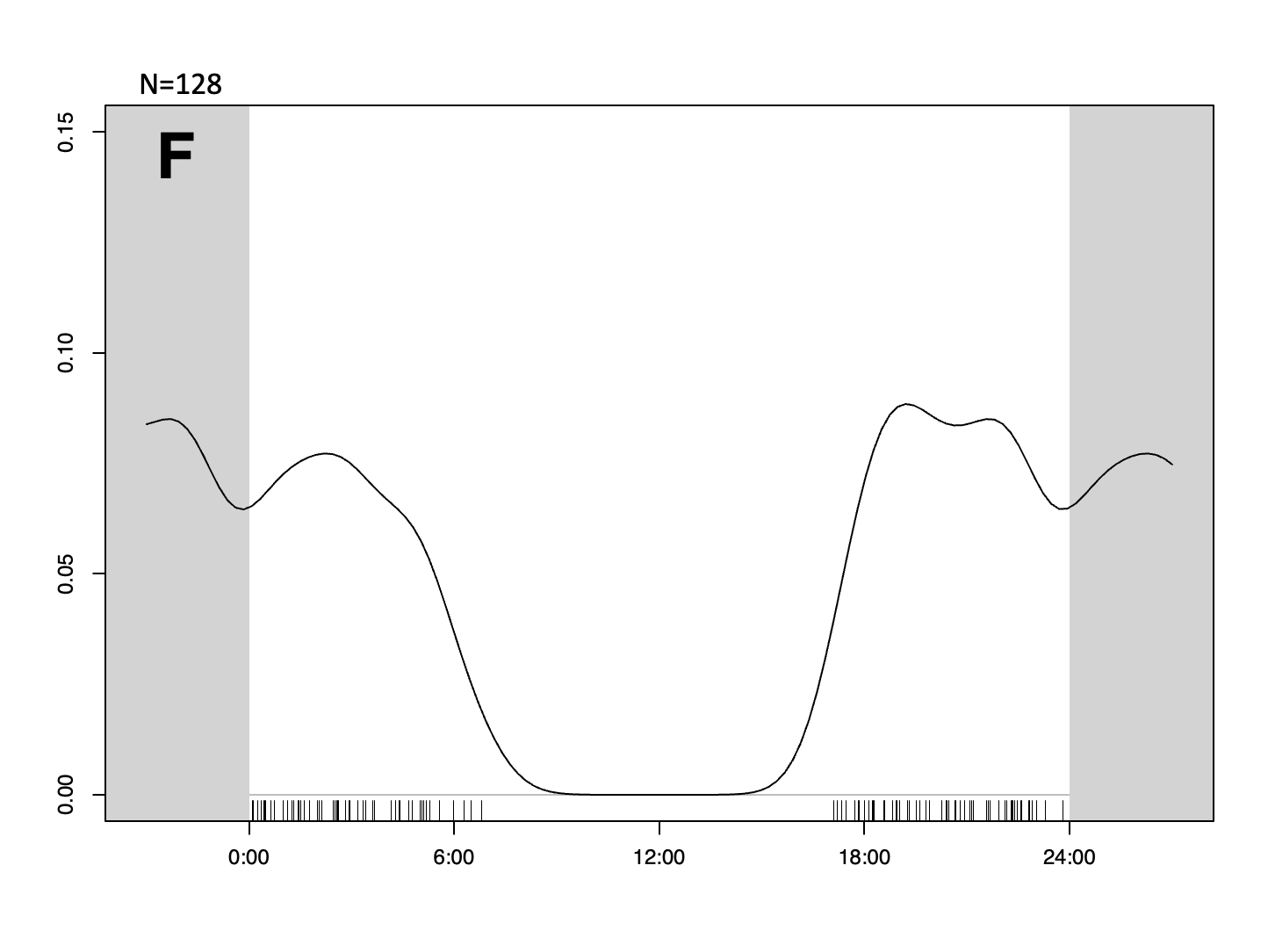

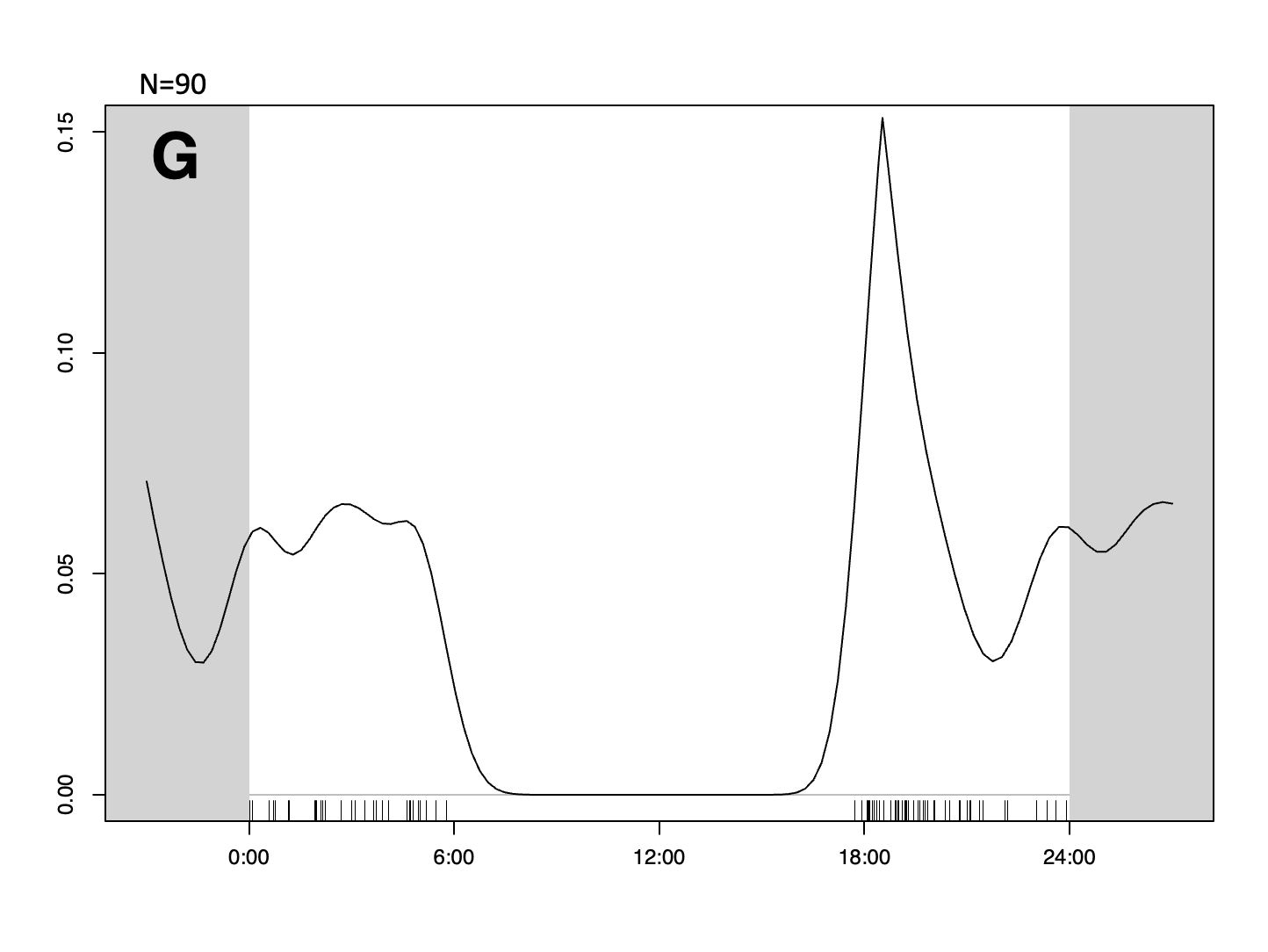

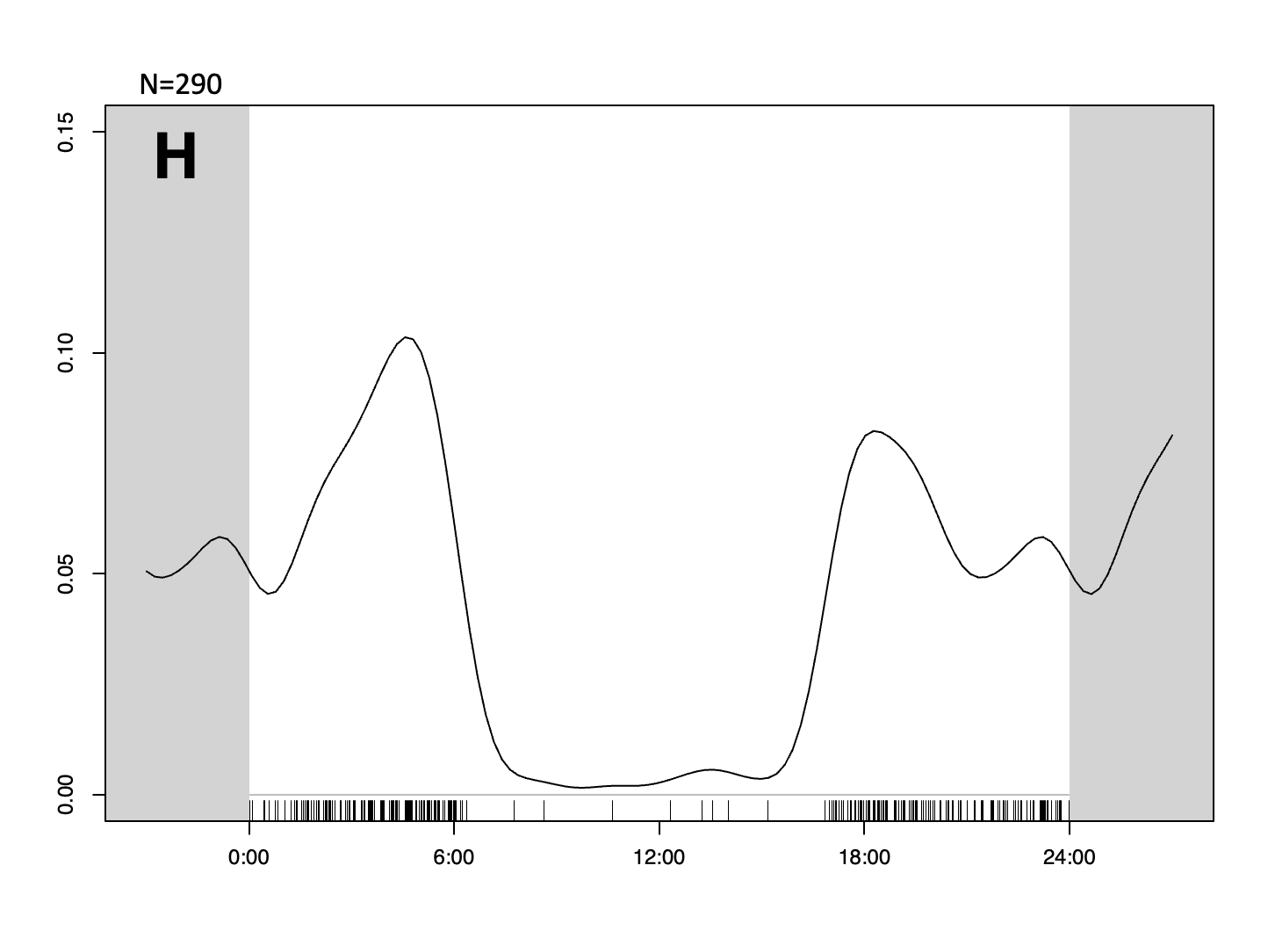


**Spring**


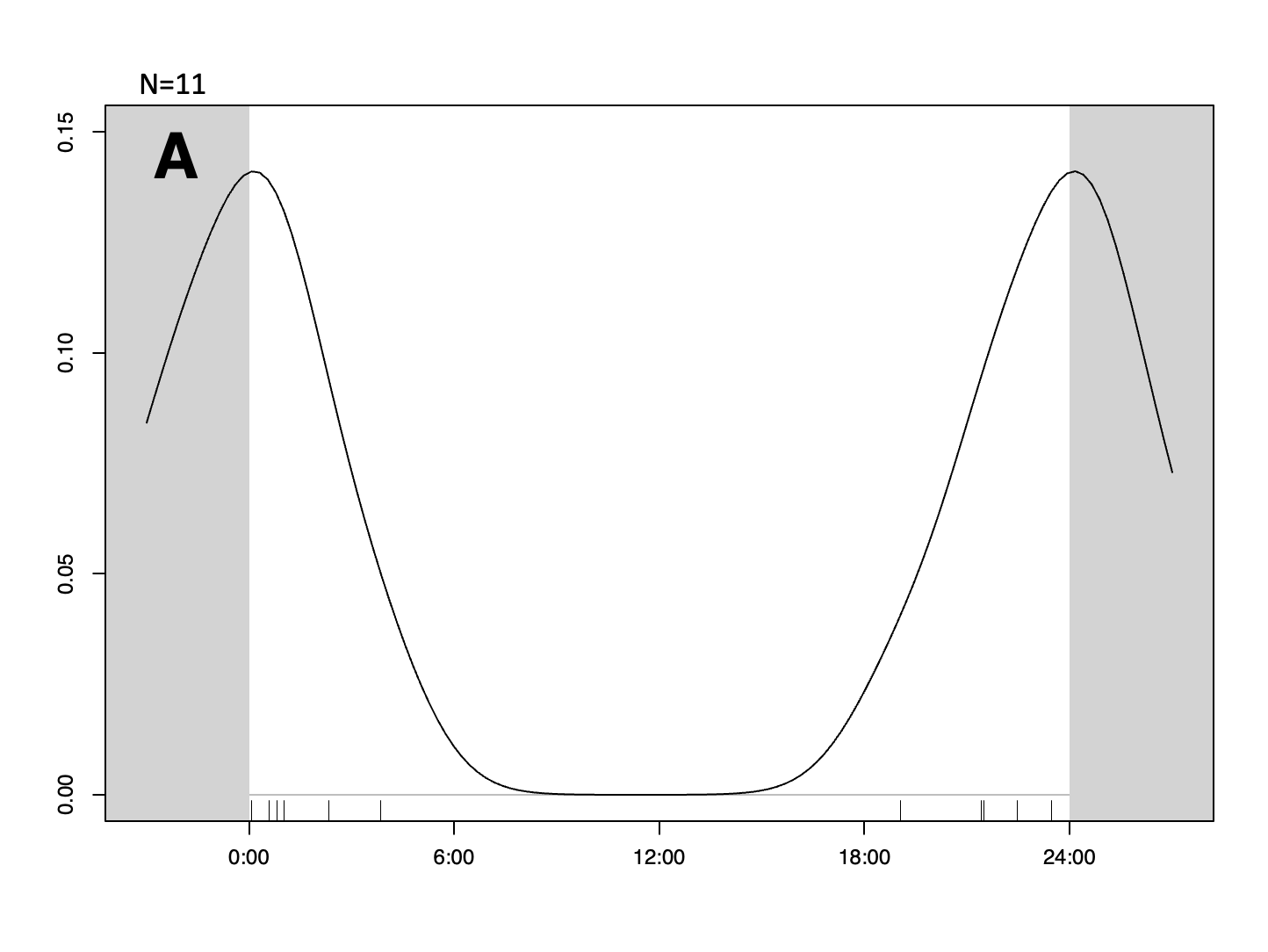

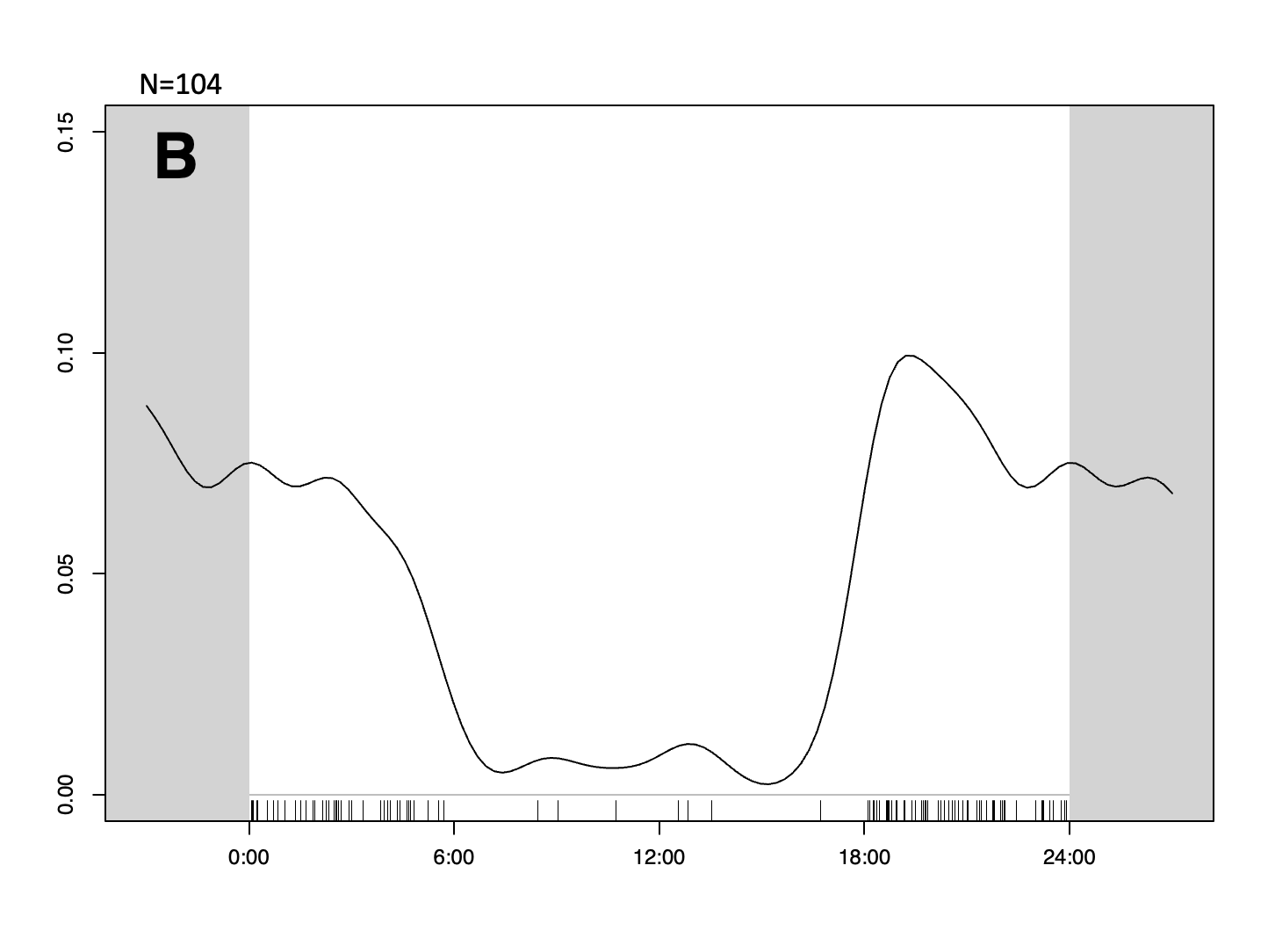

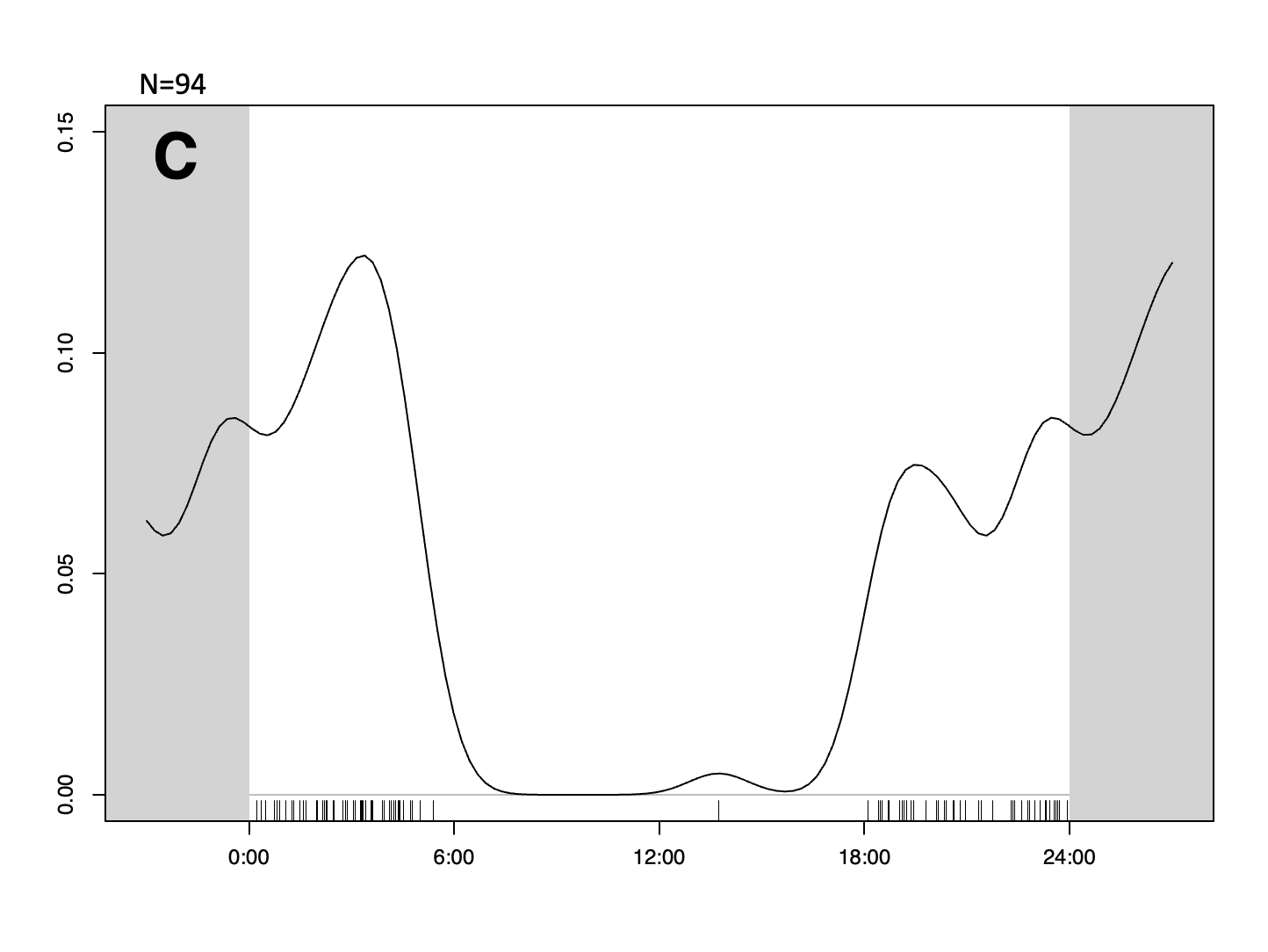

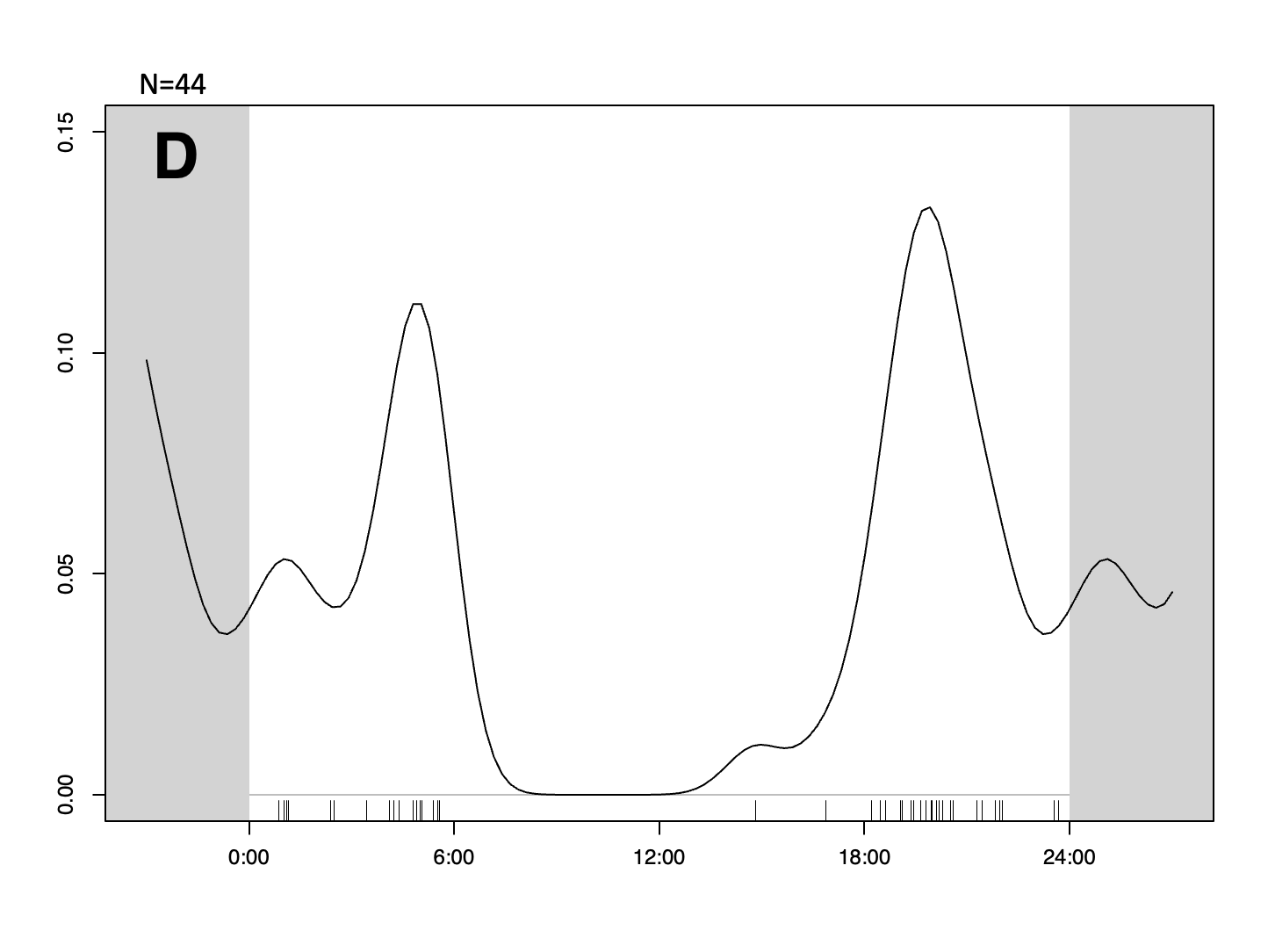

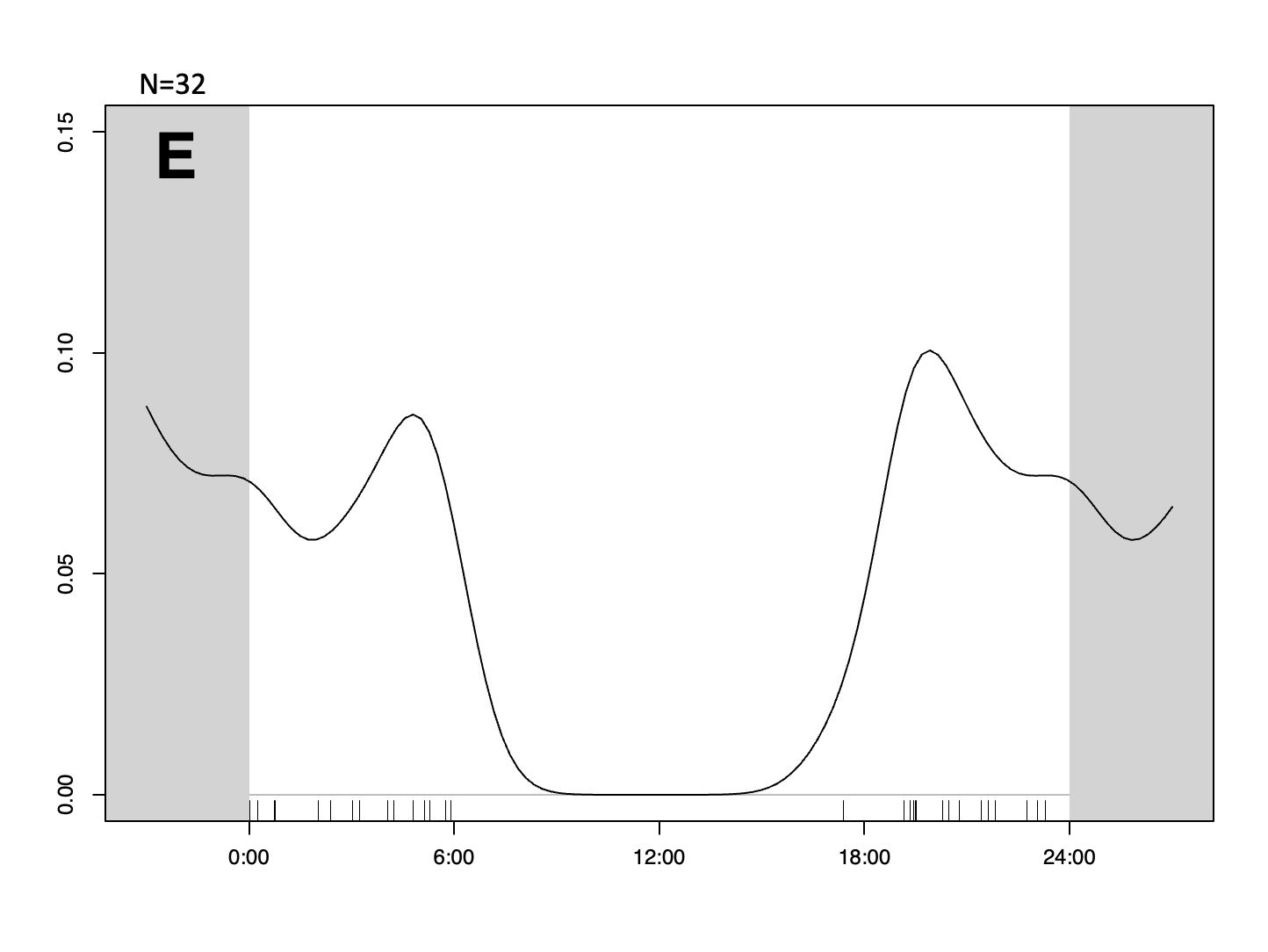

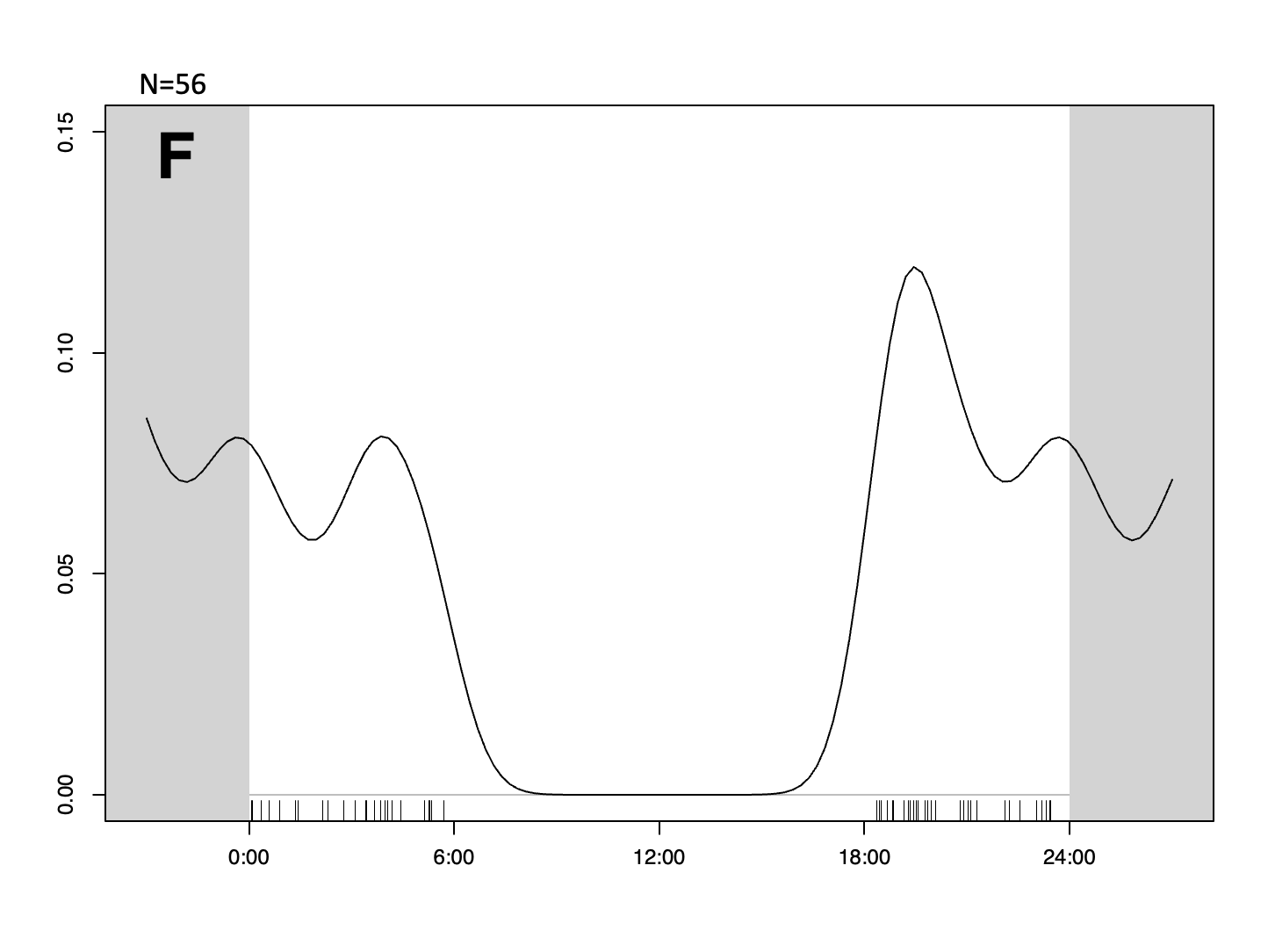


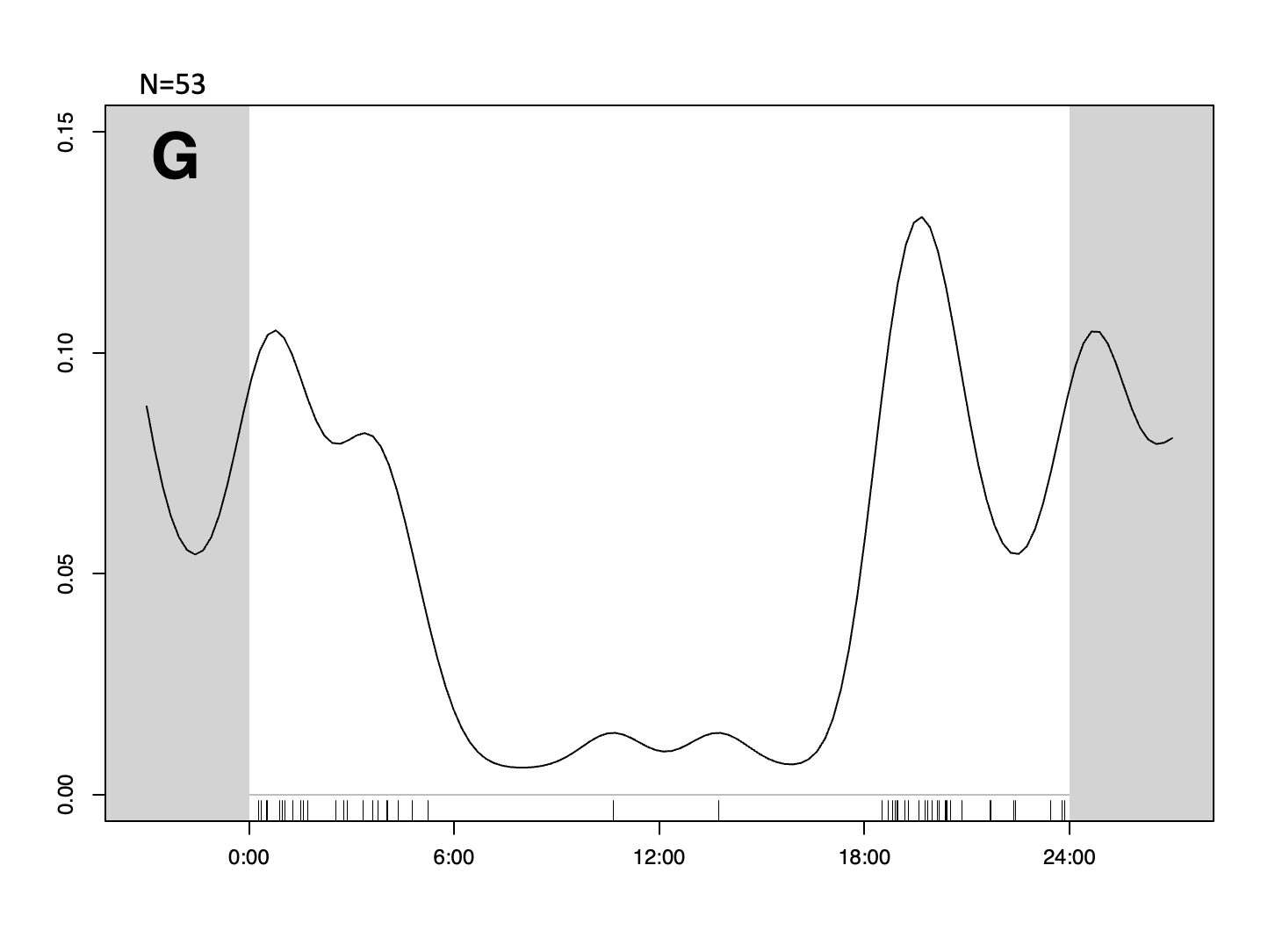

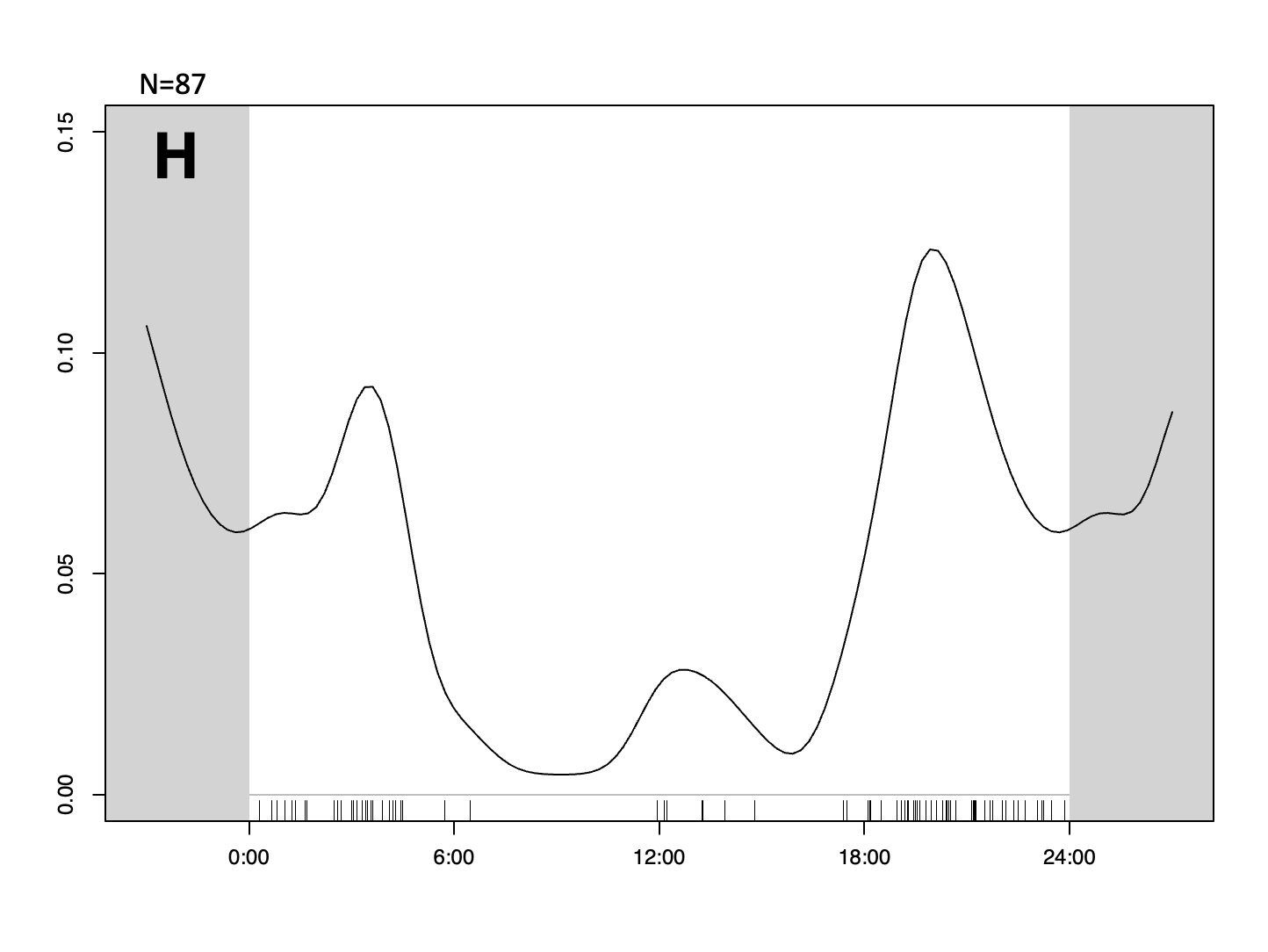


**Summer**


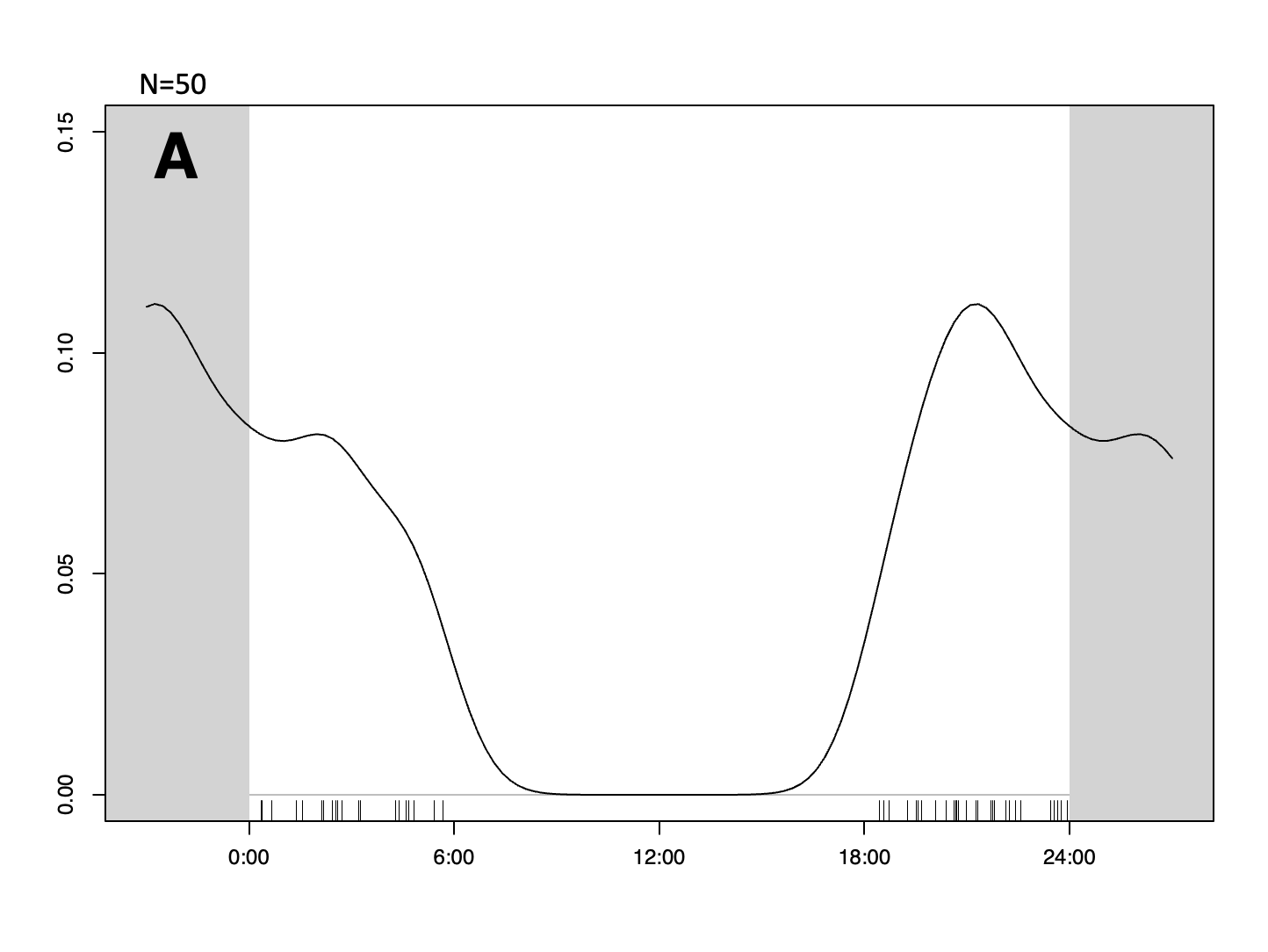

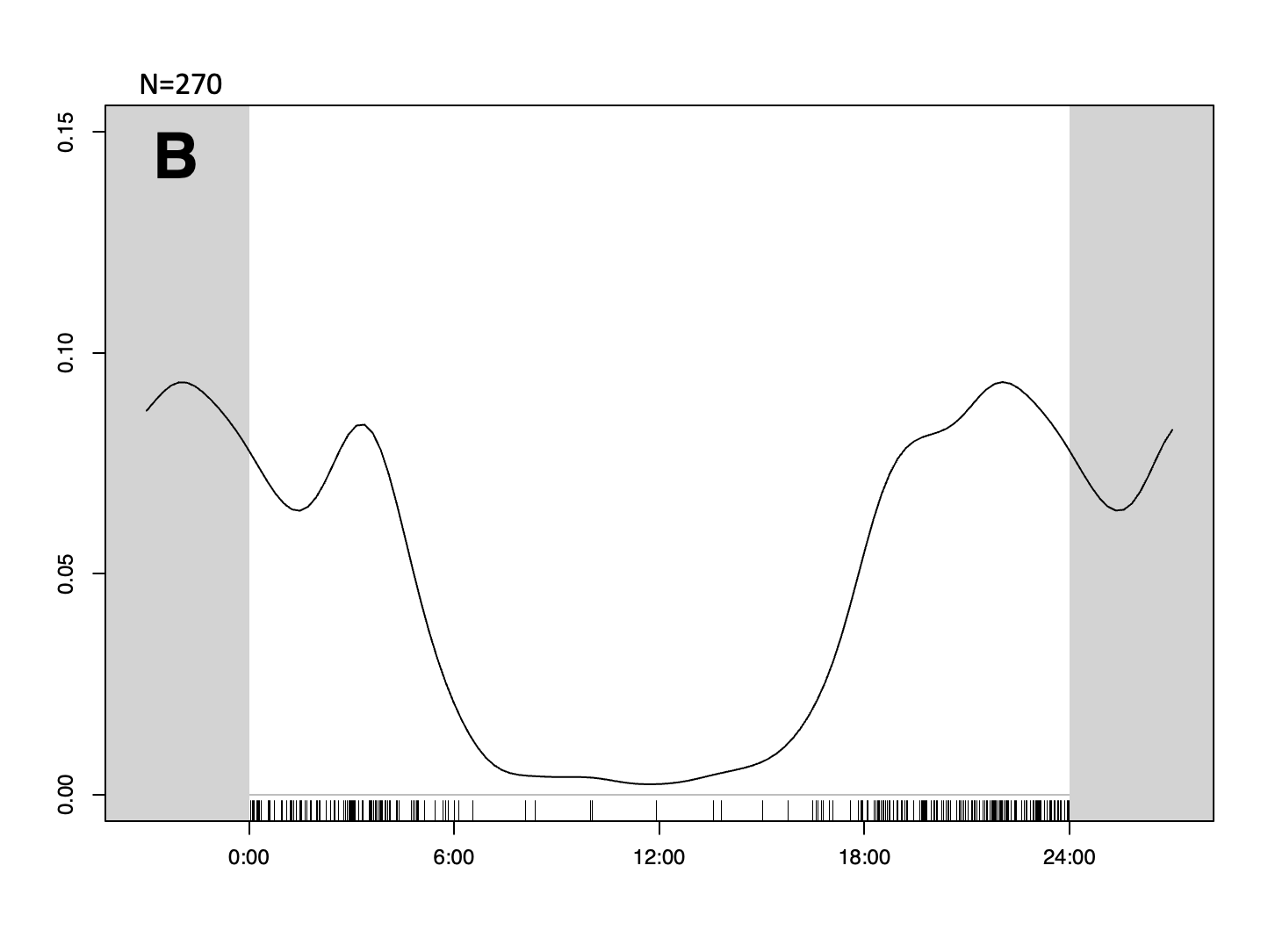

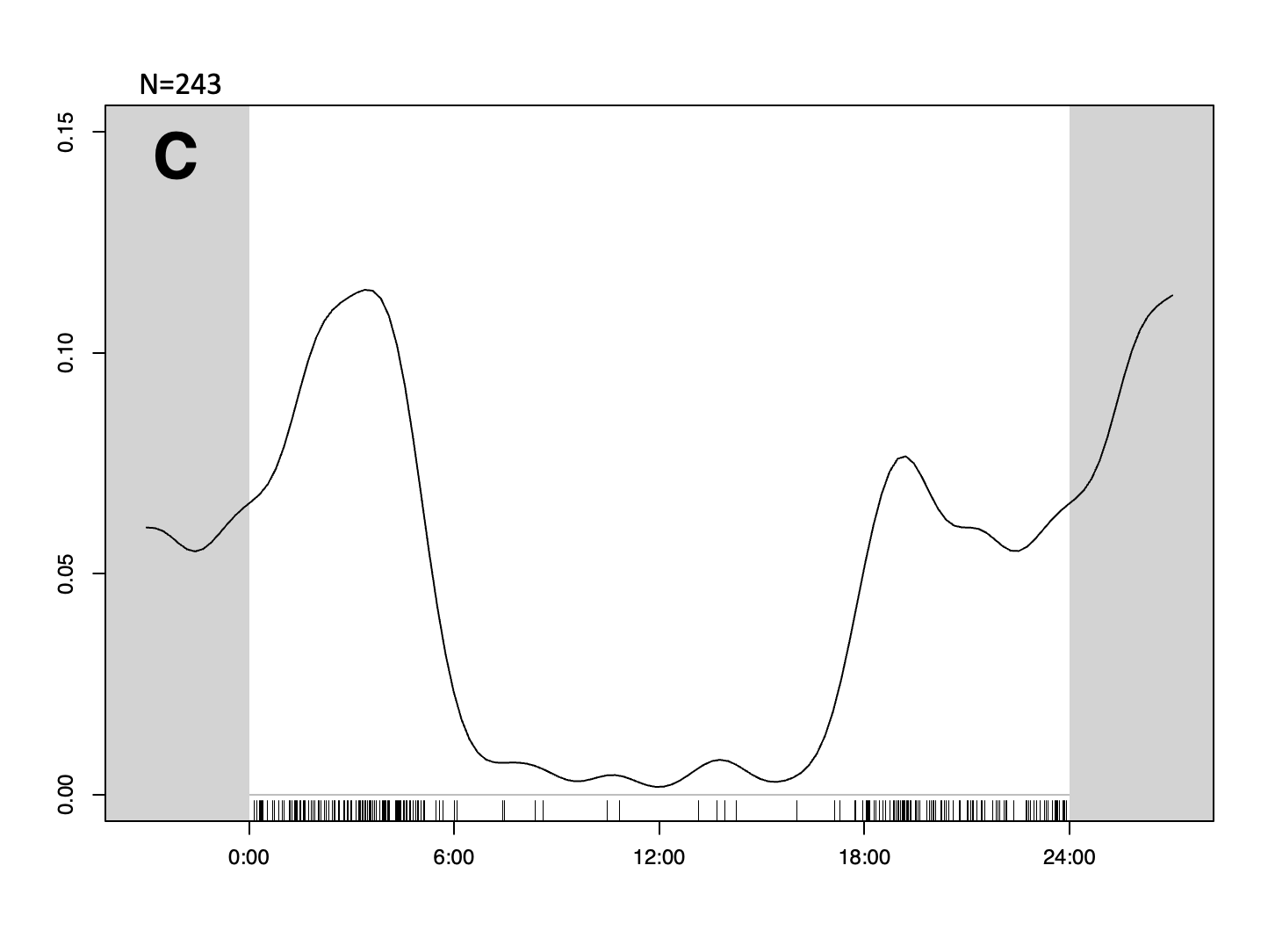

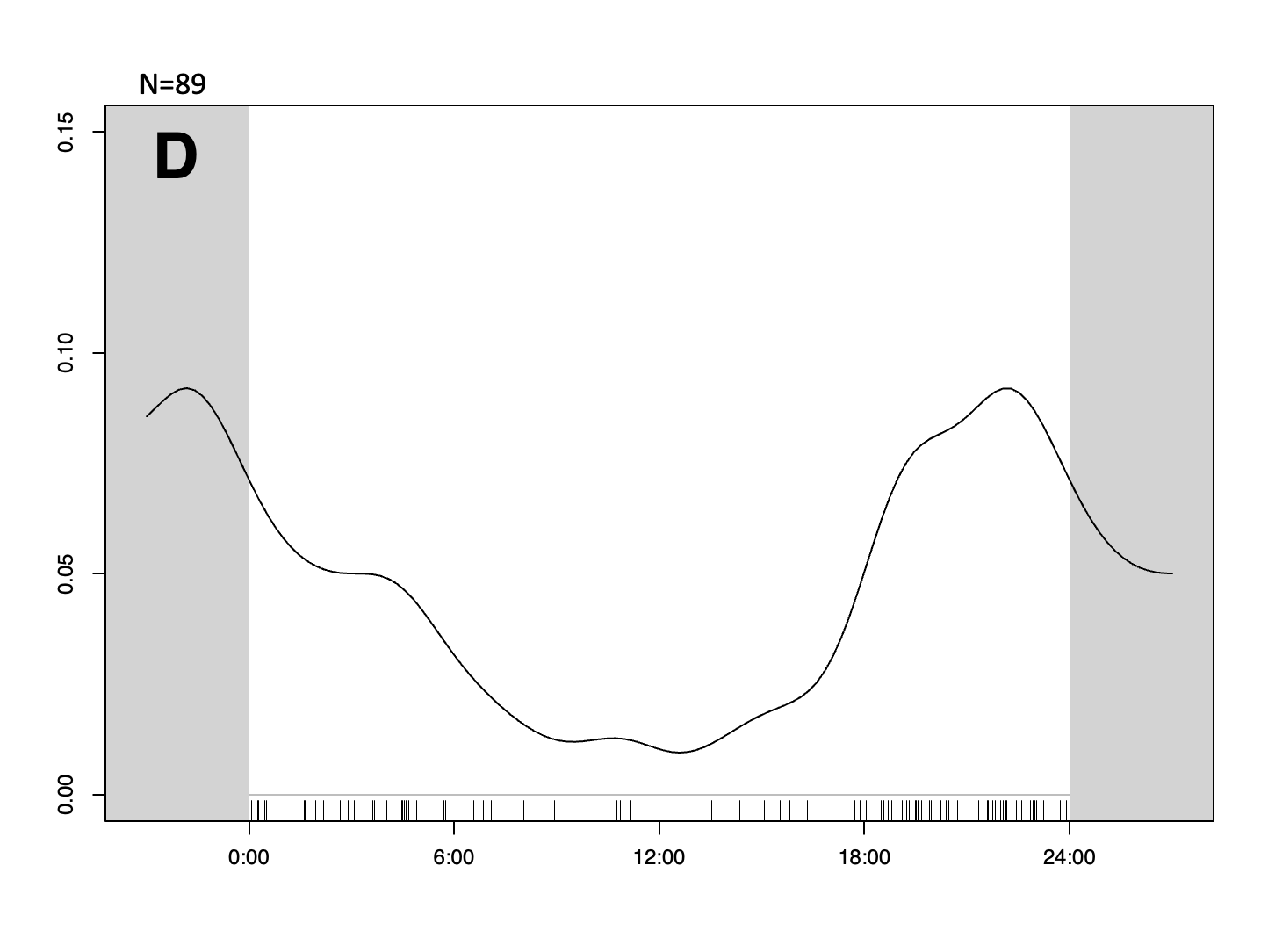

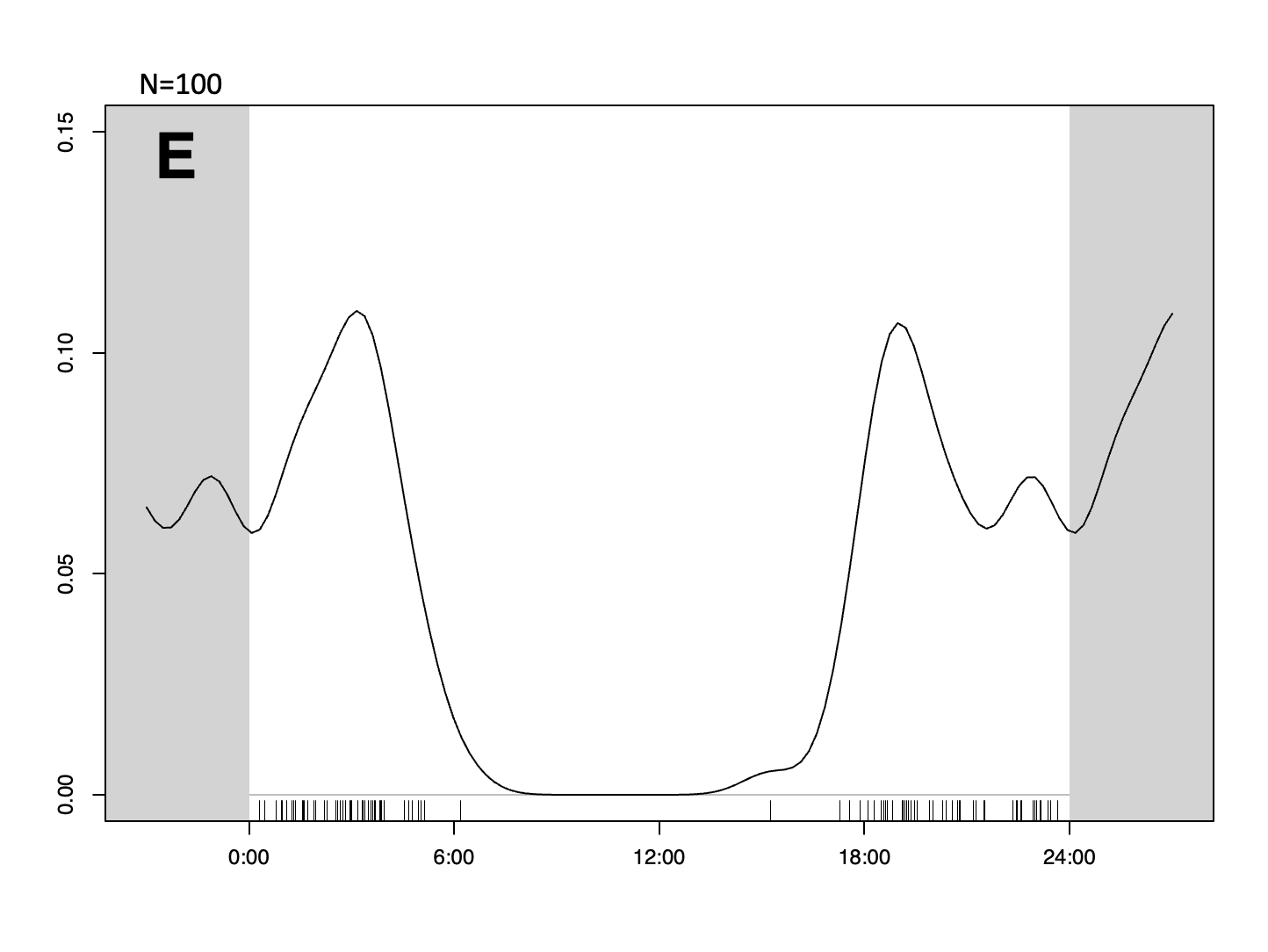

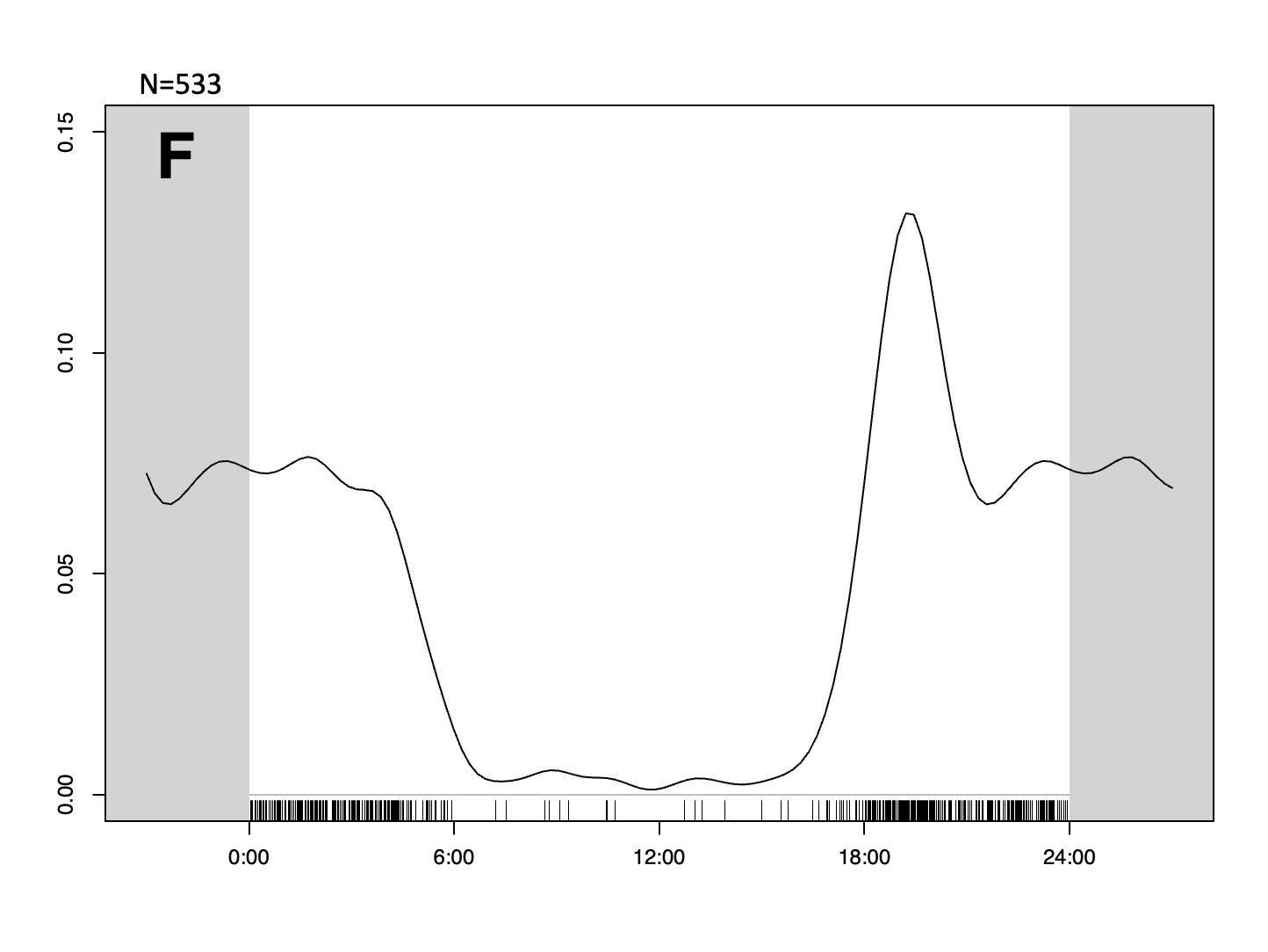

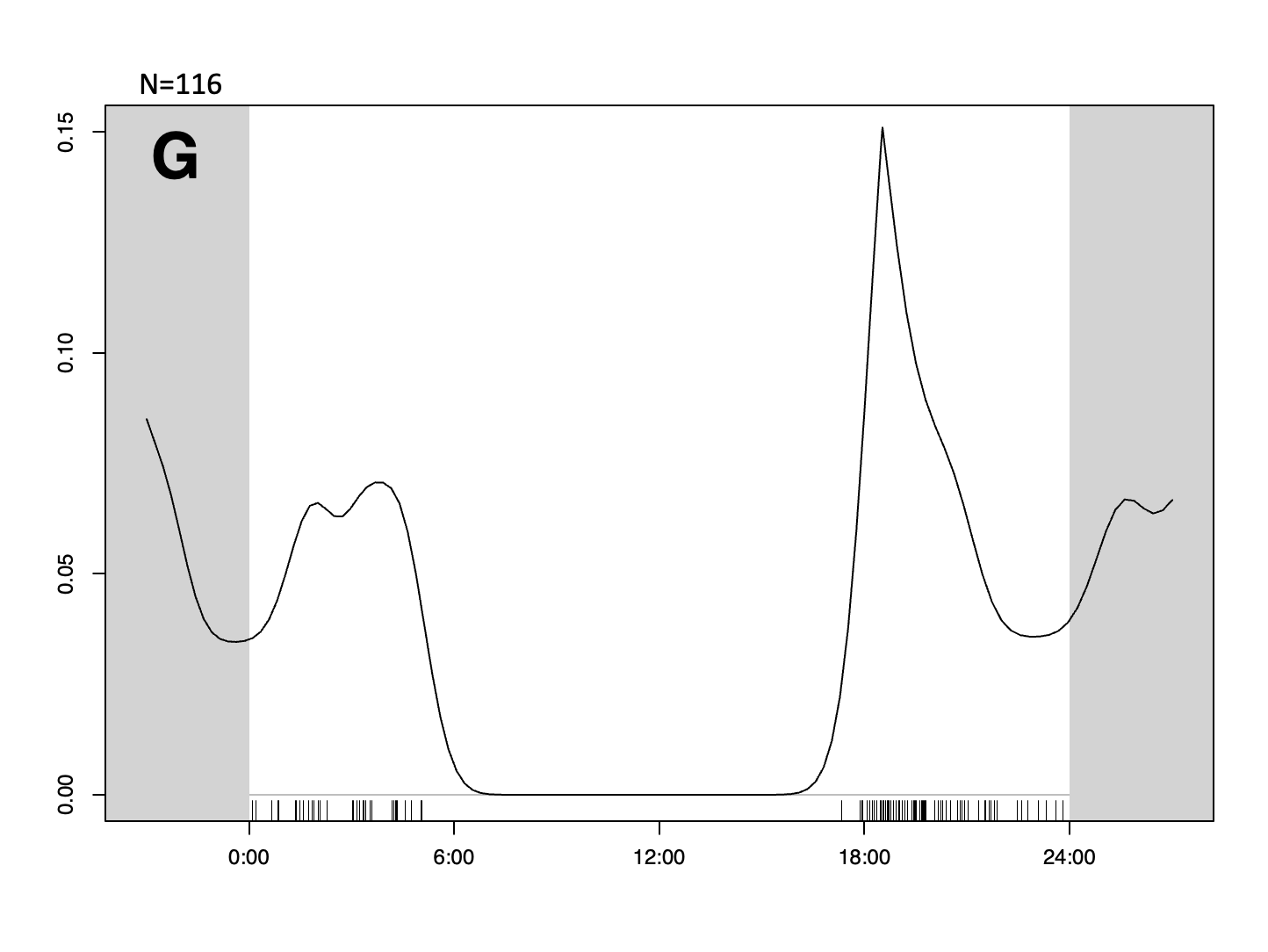

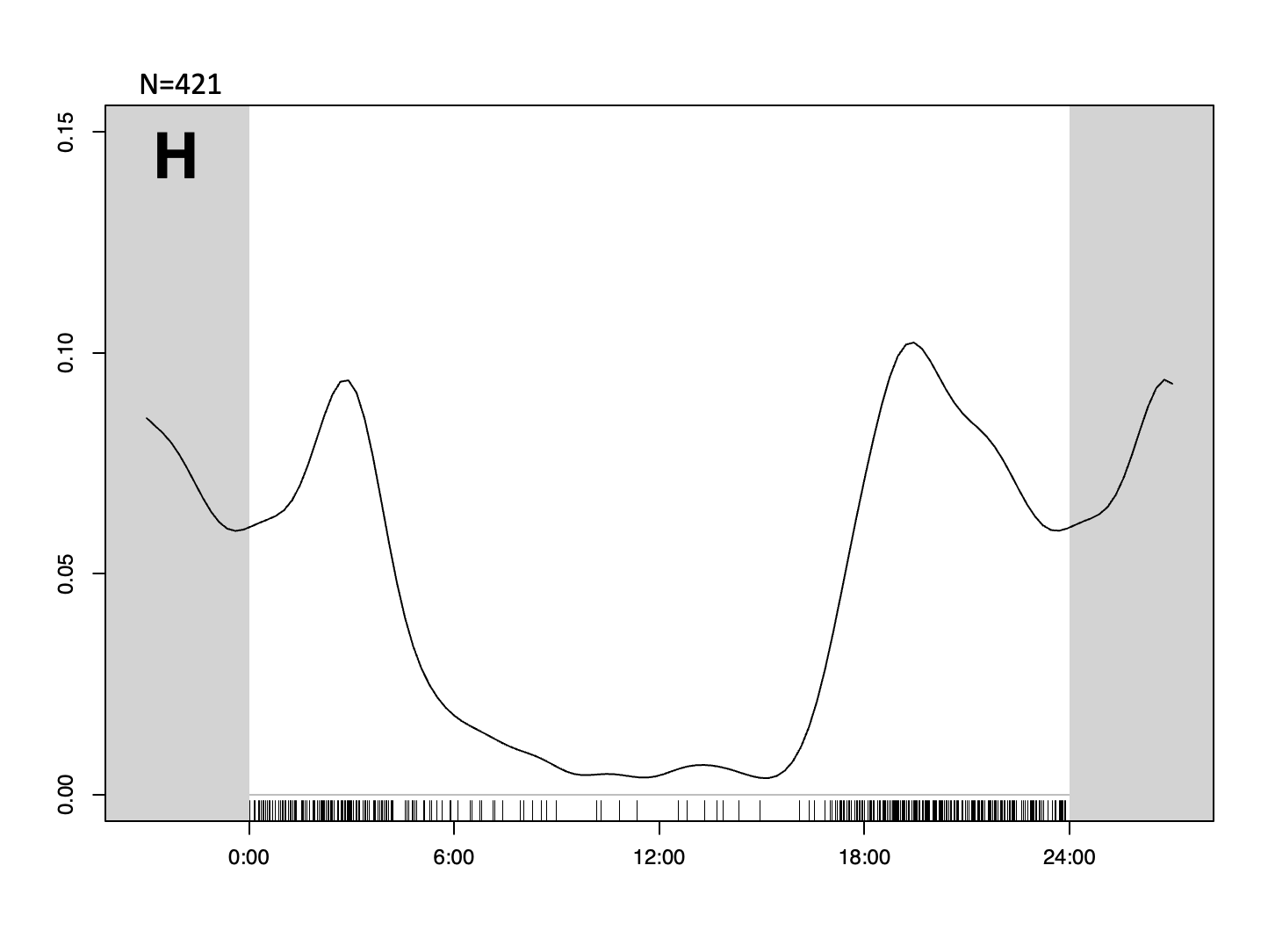

Supplement: Supplementary file 1 — Figure S1: Estimation of the diurnal activity of raccoon dogs in each camera by season and survey site using kernel density estimation. [file ECE3-15-e71966-s001.docx]
